# *Toxoplasma gondii* inhibits the expression of autophagy-related genes through AKT-dependent inactivation of the transcription factor FOXO3a

**DOI:** 10.1128/mbio.00795-23

**Published:** 2023-06-30

**Authors:** Andres Felipe Diez, Louis-Philippe Leroux, Sophie Chagneau, Alexandra Plouffe, Mackenzie Gold, Visnu Chaparro, Maritza Jaramillo

**Affiliations:** 1 Institut National de la Recherche Scientifique (INRS)—Centre Armand-Frappier Santé Biotechnologie (AFSB), Laval, Québec, Canada; University of Arizona, Tucson, Arizona, USA

**Keywords:** *Toxoplasma gondii*, FOXO3a, autophagy, AKT, host-pathogen interactions, transcriptional regulation, host response

## Abstract

The intracellular parasite *Toxoplasma gondii* induces host AKT activation to prevent autophagy-mediated clearance; however, the molecular underpinnings are not fully understood. Autophagy can be negatively regulated through AKT-sensitive phosphorylation and nuclear export of the transcription factor Forkhead box O3a (FOXO3a). Using a combination of pharmacological and genetic approaches, herein we investigated whether *T. gondii* hinders host autophagy through AKT-dependent inactivation of FOXO3a. We found that infection by type I and II strains of *T. gondii* promotes gradual and sustained AKT-dependent phosphorylation of FOXO3a at residues S253 and T32 in human foreskin fibroblasts (HFF) and murine 3T3 fibroblasts. Mechanistically, AKT-sensitive phosphorylation of FOXO3a by *T. gondii* required live infection and the activity of PI3K but was independent of the plasma membrane receptor EGFR and the kinase PKCα. Phosphorylation of FOXO3a at AKT-sensitive residues was paralleled by its nuclear exclusion in *T. gondii*-infected HFF. Importantly, the parasite was unable to drive cytoplasmic localization of FOXO3a upon pharmacological blockade of AKT or overexpression of an AKT-insensitive mutant form of FOXO3a. Transcription of a subset of *bona fide* autophagy-related targets of FOXO3a was reduced during *T. gondii* infection in an AKT-dependent fashion. However, parasite-directed repression of autophagy-related genes was AKT-resistant in cells deficient in FOXO3a. Consistent with this, *T. gondii* failed to inhibit the recruitment of acidic organelles and LC3, an autophagy marker, to the parasitophorous vacuole upon chemically or genetically induced nuclear retention of FOXO3a. In all, we provide evidence that *T. gondii* suppresses FOXO3a-regulated transcriptional programs to prevent autophagy-mediated killing.

**IMPORTANCE:**

The parasite *Toxoplasma gondii* is the etiological agent of toxoplasmosis, an opportunistic infection commonly transmitted by ingestion of contaminated food or water. To date, no effective vaccines in humans have been developed and no promising drugs are available to treat chronic infection or prevent congenital infection. *T. gondii* targets numerous host cell processes to establish a favorable replicative niche. Of note, *T. gondii* activates the host AKT signaling pathway to prevent autophagy-mediated killing. Herein, we report that *T. gondii* inhibits FOXO3a, a transcription factor that regulates the expression of autophagy-related genes, through AKT-dependent phosphorylation. The parasite’s ability to block the recruitment of the autophagy machinery to the parasitophorous vacuole is impeded upon pharmacological inhibition of AKT or overexpression of an AKT-insensitive form of FOXO3a. Thus, our study provides greater granularity in the role of FOXO3a during infection and reinforces the potential of targeting autophagy as a therapeutic strategy against *T. gondii*.

## INTRODUCTION

*Toxoplasma gondii*, the etiologic agent of toxoplasmosis, is an intracellular protozoan parasite that invades virtually any nucleated cell and infects a wide variety of warm-blooded vertebrate hosts, including humans, cats, and mice (1). It is estimated that about 30–50% of the world population is seropositive for *T. gondii* (2). The infection can be acute, chronic, or latent (3); however, symptoms, or lack thereof, at the time of infection do not predict disease manifestation later in life (1). Toxoplasmosis is generally asymptomatic, but reactivation of encysted parasites can lead to life-threatening consequences in immunocompromised individuals and cause abortions or birth defects if contracted during pregnancy (4). Yet, no effective human vaccines have been developed (1), and despite the development of new experimental drugs, none of them have been approved to prevent congenital infection while minimizing teratogenic effects (4). Thus, toxoplasmosis constitutes a serious public health concern worldwide (3).

To establish a safe replicative niche, *T. gondii* forms a non-fusogenic parasitophorous vacuole (PV) that facilitates nutrient acquisition while preventing contact with host cytoplasmic components that could trigger parasite destruction (5, 6). In addition, *T. gondii* targets host cell signaling pathways and transcription factors to evade antimicrobial responses (7, 8). Autophagy is a highly conserved homeostatic process that allows cells to degrade and recycle damaged cytosolic components; however, it can also be upregulated in response to stressors such as nutrient deprivation or infection (9). Hence, autophagy constitutes a central mechanism of host defense against intracellular pathogens, including *T. gondii* (9, 10). There are four main types of autophagy: autophagosome-mediated macroautophagy, microautophagy, chaperone-mediated autophagy, and non-canonical autophagy (9). Macroautophagy (hereinafter referred to as autophagy) inhibits *T. gondii* infection through the formation of a PV-containing autophagosome and its fusion with the lysosome (11). In addition, the PV can also be targeted *via* autophagosome-independent processes orchestrated by autophagy proteins in IFN-γ-activated cells (12).

The host cell autophagic response against *T. gondii* is mainly triggered by CD40- and IFN-γ-dependent signals that induce the expression and activation of autophagy-related proteins (e.g., ULK1, Beclin-1, and LC3), a process that culminates with the destruction of the parasite by lysosomal enzymes (13, 14). Accumulating evidence indicates that activation of the host serine/threonine kinase AKT constitutes one of the strategies developed by *T. gondii* to avoid autophagy-mediated killing (11, 15, 16). In this regard, it was reported that early and prolonged activation of EGF receptor (EGFR)-dependent AKT phosphorylation by *T. gondii* was required to prevent accumulation of autophagosome and lysosome components around the PV (i.e., LC3 and LAMP-1, respectively) and reduce parasite replication (15, 16). Of note, genetic ablation of ULK1, Beclin-1, or ATG7 hampered parasite elimination upon pharmacological blockade of EGFR, PI3K, or AKT activity (15, 16), hinting at regulation of autophagy-related proteins during *T. gondii* infection. However, the downstream targets of AKT that are responsible for *T. gondii*-driven repression of the host autophagy machinery are yet to be identified.

Regulation of autophagosome-mediated autophagy *via* the PI3K-AKT pathway is accomplished, in part, through the repression of the transcription factor Forkhead box O3a (FOXO3a; formerly Forkhead In Rhabdomyosarcoma-Like 1, FKHRL1) (17 - 19). FOXO3a is a core regulator of cellular and tissue homeostasis (e.g., cell cycle, proteostasis, proliferation, stem cell maintenance, longevity, and fertility), but it also functions as a critical modulator of stress responses (e.g., nutritional, energetic, oxidative, and genotoxic stress) (20 - 23). The subcellular distribution, stability, and transcriptional activity of FOXO3a are mainly regulated through post-translational modifications, namely, phosphorylation, acetylation, ubiquitylation, and methylation (22, 24). AKT phosphorylates FOXO3a at three highly conserved residues (i.e., S253, T32, and S315) (25). Phosphorylation of cytoplasmic FOXO3a at AKT-sensitive residues induces binding of the chaperone protein 14-3-3, which prevents FOXO3a from entering the nucleus by masking the nuclear localization signal (25). Conversely, when AKT-dependent phosphorylation occurs in the nucleus, it exposes FOXO3 nuclear export signal, thereby promoting its translocation to the cytosolic compartment (22, 26). In line with its central role in proteostasis and cellular stress responses, FOXO3a has emerged as a key transcriptional regulator of autophagy in healthy and diseased states by transactivating genes that control the formation of autophagosomes and their fusion with lysosomes (17, 19, 23, 27, 28).

Dysregulated FOXO3a activity has been detected in multiple pathologies including various types of cancers, neurodegenerative diseases, and muscle dystrophy (29, 30). Moreover, FOXO3a activity has been associated with either host protective or pathogenic roles during viral and bacterial infections (e.g., LCMV, HIV, rhinovirus, *Mycobacterium tuberculosis*, and *Citrobacter rodentium*) (31 - 35). For instance, FOXO3a promotes type I IFN antiviral innate immune responses while limiting tissue damage during rhinovirus infection (31). Similarly, FOXO3a confers protection against *M. tuberculosis* by inducing macrophage polarization toward an M1 (i.e., pro-inflammatory) phenotype (32). In stark contrast, FOXO3a activity is linked to the inhibition of T cell-mediated adaptive immune responses during HIV and LCMV infections (33, 35). Interestingly, it was recently reported that nuclear FOXO3a levels decrease in *T. gondii*-infected murine macrophages (36). However, the role and the regulation of FOXO3a during *T. gondii* infection have yet to be investigated. Here, we report that *T. gondii* hijacks the PI3K-AKT pathway to suppress autophagy-related transcriptional programs under the control of FOXO3a, thereby hindering the activation of the host autophagic response against the parasite.

## RESULTS

### *Toxoplasma gondii* induces AKT-sensitive phosphorylation of host FOXO3a

**Fig 1:**
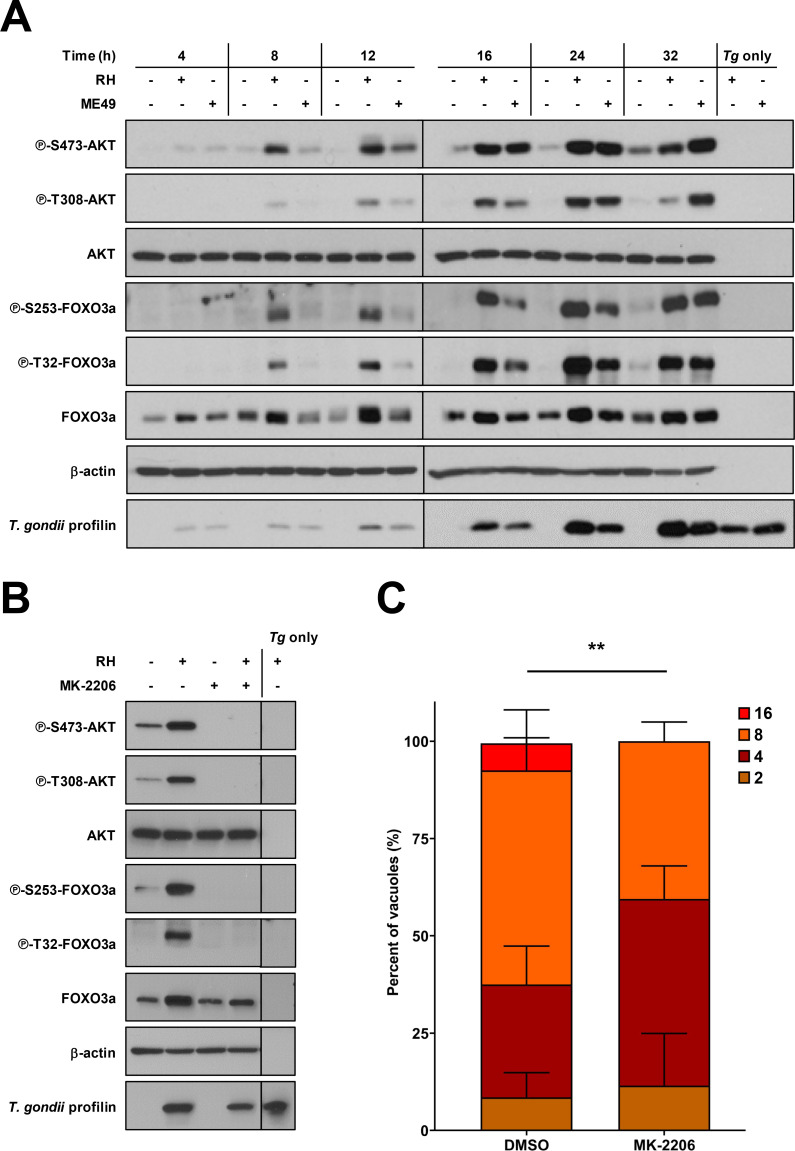
*T. gondii* induces phosphorylation of host FOXO3a and promotes its own replication within HFF in an AKT-dependent fashion. (**A**) HFF cultures were inoculated with either RH or ME49 *T. gondii* tachyzoites or left uninfected for the indicated times and then processed for Western blot analyses. (**B, C**) HFF cultures were pretreated with 2 µM MK-2206 or an equivalent volume of vehicle (i.e., DMSO) for 1 h and then inoculated with RH *T. gondii* parasites. Cells were cultured up to 32 h following infection in the presence or absence of MK-2206. (**A, B**) Phosphorylation and expression levels of indicated proteins were monitored by Western blotting. Total amounts of β-actin were used as a loading control, and an antibody raised against *T. gondii* profilin-like protein was used to assess infection of HFF cultures. Total protein extracts from extracellular tachyzoites (*Tg* only) were used to control for any cross-reactivity of the antibodies against *T. gondii* proteins. Data are representative of at least three independent experiments (i.e., performed on different days). (**C**) Cultures were fixed post infection and then processed for epifluorescence microscopy analyses. The number of parasites per PV in at least 50 infected cells in different fields of view was enumerated. Data collected from two independent experiments were compiled. **P < 0.01.

The subcellular localization and transcriptional activity of FOXO3a are tightly controlled through post-translational modifications, including AKT-dependent phosphorylation (22, 24, 25). *T. gondii* infection leads to the activation of AKT signaling (11, 15, 16); hence, we postulated that *T. gondii* modulates FOXO3a phosphorylation in an AKT-dependent fashion. To address this, we monitored changes in the phosphorylation status of AKT and FOXO3a in human foreskin fibroblasts (HFF) infected with type I or type II *T. gondii* strains (i.e., RH and ME49, respectively) (Fig. 1A). Both strains were included to evaluate any strain-specific differences in the modulation of host-signaling pathways (37). Increased phosphorylation of AKT at residues S473 and T308 was detected more rapidly in HFF infected with RH than ME49 (8 and 12 h post-infection [hpi], respectively) but was maintained up to 32 hpi in cultures infected with either strain. Accordingly, a pronounced and sustained phosphorylation of FOXO3a at AKT-sensitive residues S253 and T32 was observed in RH- and ME49-infected HFF (Fig. 1A). Importantly, the kinetics of FOXO3a phosphorylation matched closely those observed for AKT. Parasite extracts (i.e., devoid of any host cell [“*Tg* only”]) were probed in parallel to rule out the possibility that the observed changes in signaling were due to cross-reactivity of the antibodies against parasite proteins. AKT and FOXO3a phosphorylation patterns appeared to be dependent on the parasite load since increasing MOI (multiplicity of infection) ratios led to higher phosphorylation levels as observed by Western blotting analyses (Fig. S1). Similar results were obtained in mouse fibroblast cell line 3T3 upon infection with type I and type II *T. gondii* strains (Fig. S2). To confirm that *T. gondii*-inducible phosphorylation of FOXO3a was mediated by AKT, HFF cultures were treated with MK-2206, a pan-AKT inhibitor (38), or vehicle and infected with RH *T. gondii* or left uninfected. As expected, AKT phosphorylation at S473 and T308 was abrogated in the presence of MK-2206 regardless of the infection status (Fig. 1B) without affecting host cell viability (Fig. S3). Of note, pharmacological inhibition of AKT prevented FOXO3a phosphorylation at S253 and T32 in *T. gondii*-infected cells (Fig. 1B). In line with previous reports (15, 16), *T. gondii* infection rates remained unaffected by chemical blockade of AKT activity (Fig. S4), whereas parasite replication was significantly reduced (Fig. 1C). In all, this set of experiments indicates that *T. gondii* hijacks host cell signaling to drive AKT-dependent phosphorylation of the transcription factor FOXO3a which appears to favor parasite replication.

### *Toxoplasma gondii* promotes nuclear export of host FOXO3a in an AKT-dependent fashion

**Fig 2:**
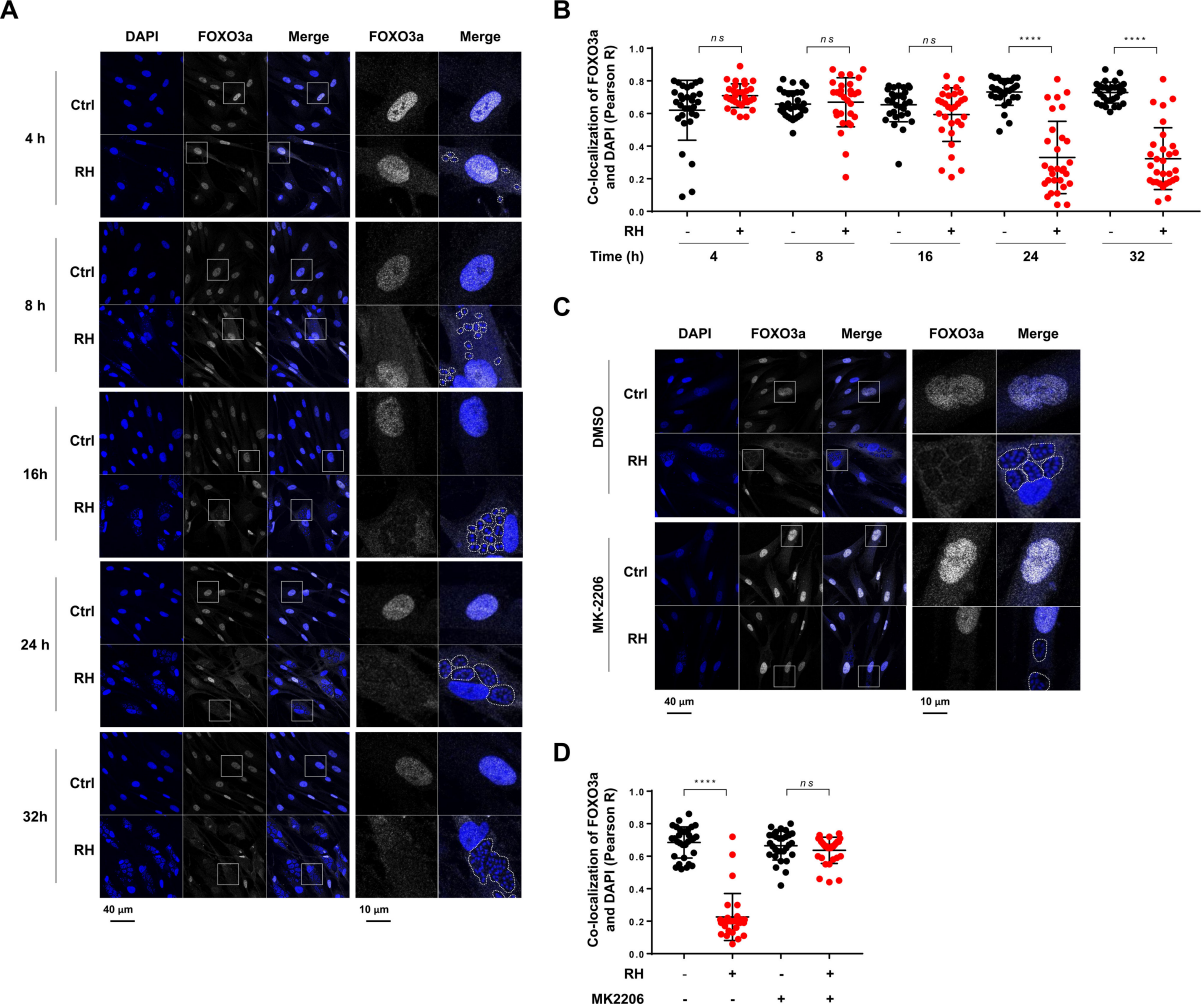
*T. gondii* infection leads to AKT-dependent FOXO3a export from the host nucleus. (**A, B**) HFF cultures were inoculated with RH *T. gondii* tachyzoites or left uninfected and fixed at the indicated times and processed for confocal immunofluorescence microscopy. (**C, D**) HFF cultures were pretreated with 2 µM MK-2206 or an equivalent volume of vehicle (i.e., DMSO) for 1 h and then inoculated with RH *T. gondii* parasites. Cells were cultured up to 32 h following infection in the presence and absence of MK-2206. (**A, C**) Samples were stained with DAPI (shown in blue), used as a nuclear marker, and for total FOXO3a (shown in white). Images are representative of at least two independent experiments. Original magnification (left panels) and four times enlarged insets (right panels). Parasitophorous vacuoles (PVs) are outlined with dashed lines to indicate the presence of parasites within infected cells. (**B, D**) Co-localization of FOXO3a and DAPI was quantified using the Pearson *R* coefficient. Data are compilated from two independent experiments (*n* = 2) in which at least 25 cells were analyzed in different fields of view. Each data point represents the Pearson *R* coefficient of a single cell. ****P < 0.0001; ns, not significant.

Given that AKT-dependent phosphorylation leads to either cytosolic retention or nuclear export of FOXO3a (22, 25, 26), we next set out to assess the subcellular localization of FOXO3a over the course of the infection by confocal microscopy. In uninfected HFF, FOXO3a was detected mostly in the nucleus throughout the entire time course (Fig. 2A and B) presumably due to the lack of phosphorylation of FOXO3a at AKT-sensitive residues as observed by Western blotting (Fig. 1A). Conversely, FOXO3a was gradually exported from the host nucleus upon RH infection (Fig. 2A and B) in an MOI-dependent fashion (Fig. S5). Significant differences in the subcellular localization of FOXO3a were detected 24 hpi and beyond, as evidenced by the decrease of the co-localization coefficient of FOXO3a with the nuclear staining (Fig. 2B). Similarly, nuclear exclusion of FOXO3a was observed in HFF cultures upon ME49 infection (Fig. S6). Consistent with our Western blot data on the effect of AKT inhibition on FOXO3a phosphorylation (Fig. 1B), HFF treatment with MK-2206 prevented *T. gondii*-inducible nuclear export of FOXO3a (Fig. 2C and D). Taken together, these data indicate that infection by *T. gondii* leads to AKT-sensitive FOXO3a nuclear export.

### *Toxoplasma gondii*-driven phosphorylation and nuclear export of FOXO3a require live infection and EGFR-independent PI3K-AKT signaling

**Fig 3:**
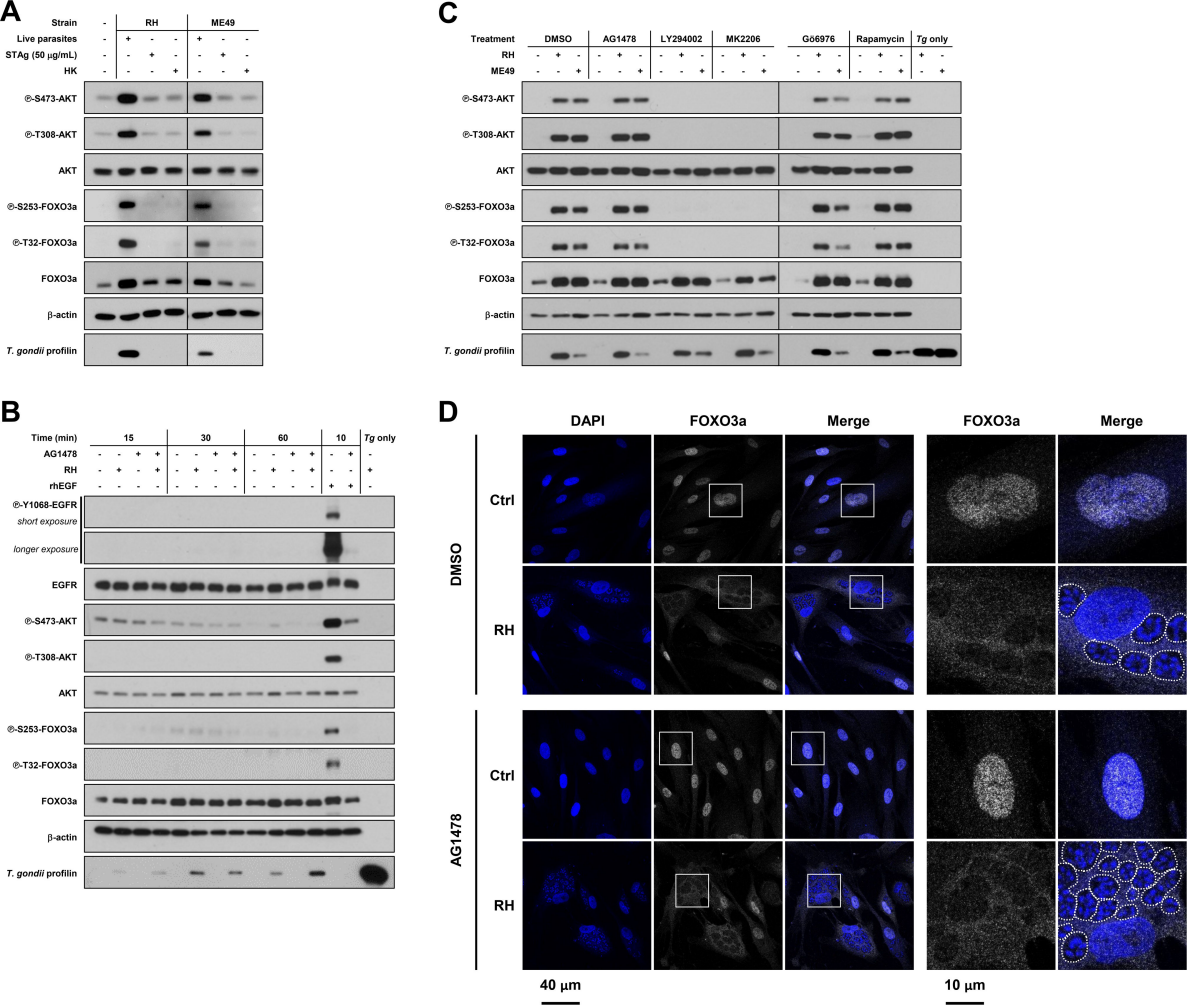
*T. gondii*-driven phosphorylation and nuclear export of FOXO3a require live infection and EGFR-independent PI3K-AKT signaling. (**A**) HFF cultures were inoculated with live or heat-killed (HK) RH or ME49 *T. gondii* tachyzoites, treated with 50 µg/mL STAg concentration, or left uninfected and untreated for 24 h. Phosphorylation and expression levels of indicated proteins were monitored by Western blotting. (**B**) HFF cultures were pre-treated with 1 µM AG1478 or an equivalent volume of vehicle (i.e., DMSO) for 1 h. Then, cultures were inoculated with RH *T. gondii* tachyzoites or left uninfected. Samples were collected at the indicated times following inoculation and processed for Western blotting analyses. As a positive control for the induced phosphorylation of EGFR, cells were treated with 100 ng/mL recombinant human EGF for 10 min. (**C**) HFF cultures were inoculated with RH or ME49 *T. gondii* tachyzoites or left uninfected for 4 h. Then, cultures were treated with the indicated inhibitors (1 µM AG1468, 20 µM LY294002, 2 µM MK-2206, 1 µM Gö6976, and 20 nM rapamycin) or an equivalent volume of DMSO (i.e., vehicle) for 20 h. Phosphorylation and expression levels of indicated proteins were monitored by Western blotting. (**D**) HFF cultures were inoculated with RH *T. gondii* tachyzoites or left uninfected for 4 h. Then, cultures were treated with 1 µM AG1468 or an equivalent volume of DMSO (i.e., vehicle) for 20 h. Samples were processed for confocal immunofluorescence microscopy. Fixed cells were stained with DAPI (shown in blue), used as a nuclear marker, and for total FOXO3a (shown in white). The data in this panel were obtained at the same time as those in Fig. 2C. DMSO images are repeated here to better depict and compare the various treatment conditions across figures. Images are representative of two independent experiments. Original magnification (left panels) and four times enlarged insets (right panels). PVs are outlined with dashed lines to indicate the presence of parasites within infected cells. Data are representative of at least two independent experiments (i.e., performed on different days).

We next sought to determine whether live infection was required to induce phosphorylation of AKT and FOXO3a. Unlike infection with live parasites, treatment of HFF cultures with soluble RH or ME49 *T. gondii* antigens (STAg) or heat-killed (HK) parasites failed to induce phosphorylation of AKT and FOXO3a (Fig. 3A). Of note, STAg did not lead to AKT and FOXO3a phosphorylation regardless of the STAg concentrations tested (Fig. S7). It was previously reported that phosphorylation of AKT upon *T. gondii* infection was, in part, regulated through the early activation of EGFR (16). To test the involvement of EGFR on the phosphorylation of AKT and FOXO3a, we pre-treated or not HFF cells with AG1418, an EGFR inhibitor (39); then inoculated cultures or not with RH *T. gondii* tachyzoites; and collected samples at early timepoints (i.e., 15, 30, and 60 min post-inoculation). Infection did not induce noticeable levels of AKT and FOXO3a phosphorylation at these timepoints (Fig. 3B). In parallel, HFF cultures were stimulated with recombinant human EGF (rhEGF) for 10 min as a positive control for EGFR activation. Treatment with rhEGF strongly induced the phosphorylation of EGFR (Y1068) as well as AKT and FOXO3a. Moreover, pre-treatment with AG1418 abrogated rhEGF-induced phosphorylation of EGFR, AKT, and FOXO3a, confirming the efficacy of the inhibitor at the tested concentration. These data indicate that *T. gondii*-driven phosphorylation of FOXO3a requires live infection and is independent of early EGFR and AKT activation.

Despite the absence of early EGFR activation during *T. gondii* infection, we assessed its role in the gradual and sustained phosphorylation of AKT and FOXO3a observed at later timepoints p.i. To do so, we first infected or not HFF cultures with either RH or ME49 *T. gondii* tachyzoites. Then, 4 h after inoculation, cultures were treated with AG1418 or vehicle and further incubated for an additional 20 h. This approach helped avoid any effects of the compound on parasite’s ability to invade host cells. Pharmacological inhibition of EGFR activity did not affect the parasite’s ability to induce the phosphorylation of AKT and FOXO3a (Fig. 3C). Confocal immunofluorescence microscopy analyses confirmed that infection by *T. gondii* still led to the nuclear export of FOXO3a despite concomitant treatment with AG1478 (Fig. 3D). In light of these results, we sought for alternative molecular mechanisms responsible for *T. gondii*-induced phosphorylation of FOXO3a. It has been previously shown that *T. gondii* can phosphorylate AKT through the activation of its upstream regulator phosphatidylinositol 3-kinase (PI3K) (16, 40 - 42). Treatment with LY294002, a potent inhibitor of PI3K (43), and MK-2206 completely inhibited AKT and FOXO3a phosphorylation (Fig. 3C). In addition to EGFR and PI3K, PKCα and mTOR have been shown to upregulate AKT activity in *T. gondii*-infected cells (15, 44). To evaluate the potential contribution of these regulators, we treated HFF cultures with either Gö6976 or rapamycin, a PKCα and an mTOR complex 1 (mTORC1) inhibitor, respectively (45, 46). These inhibitors failed to prevent infection-induced phosphorylation of AKT and FOXO3a (Fig. 3C). Of note, none of these inhibitory compounds displayed overt toxicity on HFF cells at the tested concentrations (Fig. S3). Taken together, these results suggest that the PI3K-AKT signaling axis is required to induce the phosphorylation of FOXO3a during *T. gondii* infection but independently of EGFR, PKCα, or mTORC1 activity.

### *Toxoplasma gondii* inhibits the expression of autophagy-related genes through AKT-dependent inactivation of FOXO3a

**Fig 4:**
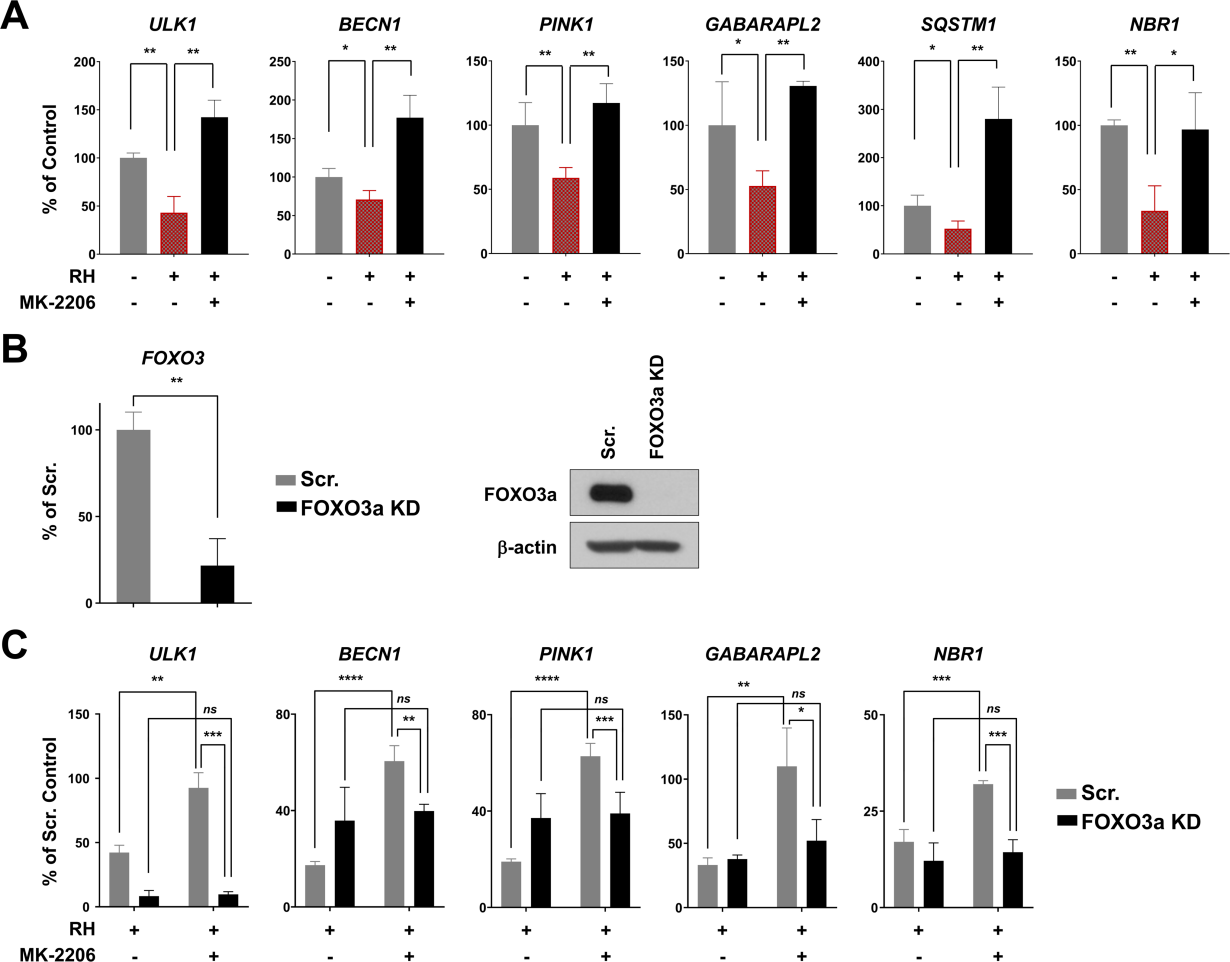
AKT-FOXO3a-sensitive transcription of autophagy-related host genes induced upon serum starvation is impeded following *T. gondii* infection. (A) HFF cultures were pretreated with 2 µM MK-2206 or an equivalent volume of vehicle (i.e., DMSO) for 1 h and then inoculated with RH *T. gondii* parasites or left uninfected. To induce autophagy, cultures were deprived of serum throughout the course of the infection (24 h). (B) Knockdown levels of *FOXO3* mRNA and FOXO3a protein in lentivirus-transduced HFF were monitored by qPCR and Western blotting, respectively, and compared to cells transduced with scrambled (Scr.) shRNA. (C) Scr. or *FOXO3* shRNA-transduced HFF cultures were pretreated with 2 µM MK-2206 or an equivalent volume of vehicle (i.e., DMSO) for 1 h and then inoculated with RH *T. gondii* parasites or left uninfected. Cultures were deprived of serum throughout the course of the infection (24 h). (A; B, left panel; and C) Relative expression of indicated genes was normalized to *ACTB* and was calculated as a percentage of (A) serum-starved DMSO-treated uninfected control (i.e., “Control”), (B) Scr. HFF cultures, or (C) serum-starved DMSO-treated uninfected Scr. HFF cultures. Data are presented as mean (SD) in technical triplicates and are representative of at least two independent experiments (i.e., performed on different days). ****P < 0.0001; ***P < 0.001; **P < 0.01; *P < 0.05; ns, not significant.

Previous reports support the notion that *T. gondii* prevents autophagy-mediated killing through AKT-dependent signaling (11, 15, 16); however, the AKT downstream effector(s) are yet to be identified. FOXO3a is an important transcription factor of autophagy-related genes (17 - 19) that is under the control of AKT (22, 25). Hence, we postulated that AKT blocks FOXO3a-dependent autophagy-related transcriptional programs during *T. gondii* infection. To test this, we induced transcription of autophagy-related genes in HFF cultures by serum starvation, a well-described approach to induce FOXO3a-dependent autophagy (47). In addition, cells were treated with MK-2206 to prevent AKT-dependent phosphorylation and nuclear export of FOXO3a. Serum starvation led to an increase of all autophagy-related transcripts monitored by RT-qPCR in uninfected HFF cells compared to control cells not deprived of serum (Fig. S8A). Of note, infection by *T. gondii* significantly inhibited starvation-induced transcription of autophagy-related genes (i.e., *ULK1*, *BECN1*, *PINK1*, *GABARAPL2*, *SQSTM1*, and *NBR1*), as compared to uninfected serum-starved HFF cultures (i.e., “Control”) (Fig. 4A and Fig. S8A, top panel). Conversely, transcription of several other autophagy-related genes was not inhibited by *T. gondii* in serum-starved HFF cells (i.e., *ATG5*, *ATG7*, *ATG12*, *ATG16L*, *BNIP3L*, and *GABARAP*) (Fig. S8A, bottom panel). Inhibition of AKT by MK-2206 treatment enhanced the expression of *ULK1*, *BECN1*, *PINK1*, *GABARAPL2*, *SQSTM1*, and *NBR1* in infected cells (Fig. 4A and Fig. S8A, top panel), highlighting the requirement of intact AKT signaling for selective transcriptional repression of autophagy-related genes by *T. gondii*.

To resolve *T. gondii*-driven repression of autophagy-related genes through AKT-dependent inactivation of FOXO3a, we generated a FOXO3a knockdown (KD) HFF cell line by shRNA targeting. In parallel, control HFF cultures were transduced to express a non-targeting scrambled (Scr.) shRNA. The efficiency of the KD of *FOXO3* and subsequent inhibition of FOXO3a protein expression were confirmed by RT-qPCR and Western blot analyses, respectively (Fig. 4B). Infection of either Scr. or FOXO3a KD HFF by *T. gondii* markedly inhibited transcription of *ULK1*, *BECN1*, *PINK1*, *GABARAPL2*, *SQSTM1*, and *NBR1* as compared to uninfected serum-starved Scr. HFF cultures (i.e., “Scrambled Control”) (Fig. 4C and Fig. S8B). In line with RT-qPCR experiments carried out in wild-type (WT) HFF (Fig. 4A and Fig. S8A, top panel), treatment with MK-2206 enhanced transcription of autophagy-related genes in *T. gondii*-infected Scr. HFF (Fig. 4C and Fig. S8B). In stark contrast, pharmacological blockade of AKT did not reverse the inhibitory effect of *T. gondii* on the expression of *ULK1*, *BECN1*, *PINK1*, *GABARAPL2,* and *NBR1* mRNAs in FOXO3a KD HFF (Fig. 4C). Interestingly, *T. gondii*-driven repression of *SQSTM1* transcription appeared to be AKT-dependent but FOXO3a-independent since AKT inhibition led to an increase in *SQSTM1* mRNA levels in both infected Scr. and FOXO3a KD HFF cultures (Fig. S8B). This set of experiments provides evidence, and *T. gondii* downregulates the expression of selected autophagy-related genes through AKT-dependent inactivation of FOXO3a transcriptional programs.

### *Toxoplasma gondii* drives AKT-sensitive nuclear export of FOXO3a to prevent targeting of the parasitophorous vacuole by the host autophagic response

**Fig 5:**
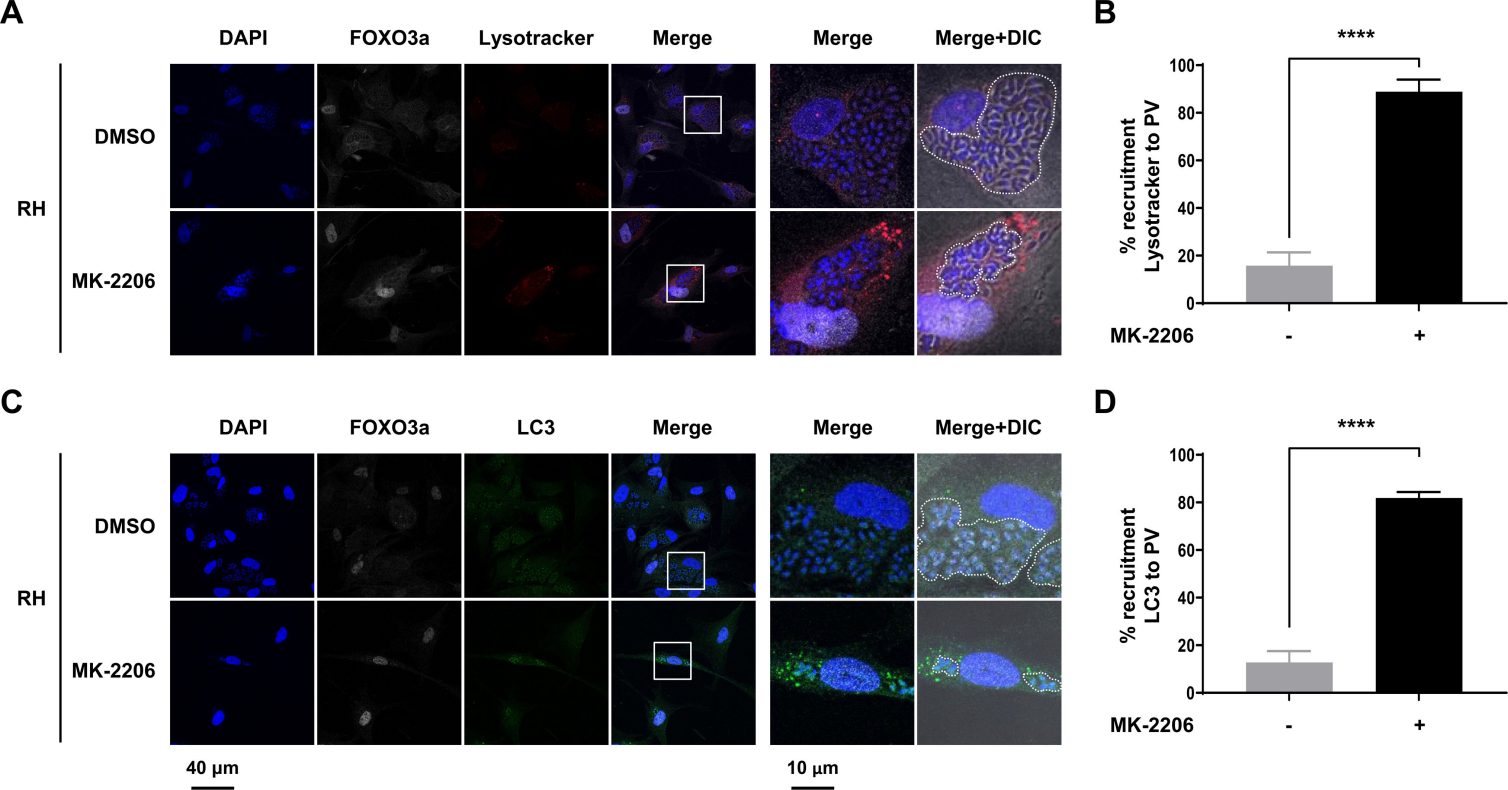
AKT inhibition leads to the recruitment of autophagolysosomal markers at the parasitophorous vacuole. (A, B) HFF cultures were treated with 2 µM MK-2206 or an equivalent volume of vehicle (i.e., DMSO) for 1 h. Cells were then inoculated with RH *T*. gondii parasites or left uninfected for 24 h. Cultures were serum-starved for the entire length of the experiment. Samples were processed for confocal immunofluorescence microscopy. As shown here, cells were stained with DAPI, for FOXO3a, and (A, B) LysoTracker Red DND-99 (2 h prior to fixation) or (C, D) LC3. Original magnification (left panels) and four times-enlarged insets (right panels). Data are representative of two biological replicates. (A, C) PVs are outlined with dashed lines to indicate the presence of parasites within infected cells. Recruitment of (B) lysosomal/autophagolysosomal structures (Lysotracker-stained) and (D) LC3 to the PV was quantified. In DMSO-treated cells, 62 and 73 PVs were analyzed for lysosomal/autophagolysosomal structure and LC3 recruitment, respectively. In MK-2206-treated cells, 37 and 43 PVs were analyzed for lysosomal/autophagolysosomal structure and LC3 recruitment, respectively. Data are presented as mean (SD) and are representative of three independent experiments (i.e., performed on different days). ****P < 0.0001.

*T. gondii* is able to block the recruitment of host phagolysosomes and autophagy effectors to the PV to avoid elimination (11, 15, 16, 48). As previously reported, this is mediated, in part, through the sustained activation of AKT (15, 16). To confirm this in our system, we infected serum-deprived HFF cultures treated or not with MK-2206 to inhibit AKT. We then stained cells with LysoTracker Red DND-99, which selectively stains acidic organelles, to monitor the presence and recruitment of lysosomal/autophagolysosomal structures. Consistent with data shown in Fig. 2C, FOXO3a was excluded from the host nucleus in infected cells but remained nuclear upon MK-2206 treatment (Fig. 5A and C). Pharmacological blockade of AKT also led to a pronounced increase in the staining intensity and recruitment of acidic structures around the PV as compared to DMSO-treated infected cells (Fig. 5A and B). In parallel, we assessed the expression and localization of LC3, a well-described autophagy effector protein (9), in these cells. While staining was relatively weak and diffused in the entire cytoplasm of infected DMSO-treated HFF cultures, punctate staining patterns for LC3 were readily observed surrounding the PV in MK-2206-treated cells (Fig. 5B and D). In line with previous reports (15, 16), these data indicate that intact AKT activity in *T. gondii*-infected cells is required to prevent autophagolysosomal targeting of the PV.

To complement our pharmacological approach, HFF cultures were transduced to express either a N-terminus Myc-tagged WT or an AKT-resistant form of FOXO3a (i.e., Triple Mutant; TM), in which AKT-sensitive residues S253, T32, and S315 are mutated to alanine (i.e., S253A, T32A, and S315A) (17, 25, 28). To ensure that the exogenous Myc-FOXO3a WT form behaved similarly to the endogenous protein *vis-à-vis* AKT-sensitive subcellular localization, we transduced HFF cells to express the Myc-FOXO3a WT form or with the Empty vector. Then, cells were cultured in nutrient-rich medium (i.e., 10% FBS) to promote nuclear export of FOXO3a under non-restrictive growth conditions and treated with MK-2206 or DMSO. Using an anti-Myc-tag-specific antibody, Myc-FOXO3a WT was readily observed in the cytoplasm of DMSO-treated cells but was predominantly nuclear upon AKT inhibition (Fig. S9A). No signal was detected in Empty vector-transduced HFF cultures, highlighting the specificity of the immunostaining (Fig. S9A). Staining cells using an anti-FOXO3a antibody revealed a similar subcellular distribution in DMSO versus MK-2006 treatment (Fig. S9B). Moreover, this latter approach confirmed higher expression of FOXO3a in Myc-FOXO3a WT transduced cells as compared to Empty vector-transduced control HFF.

**Fig 6:**
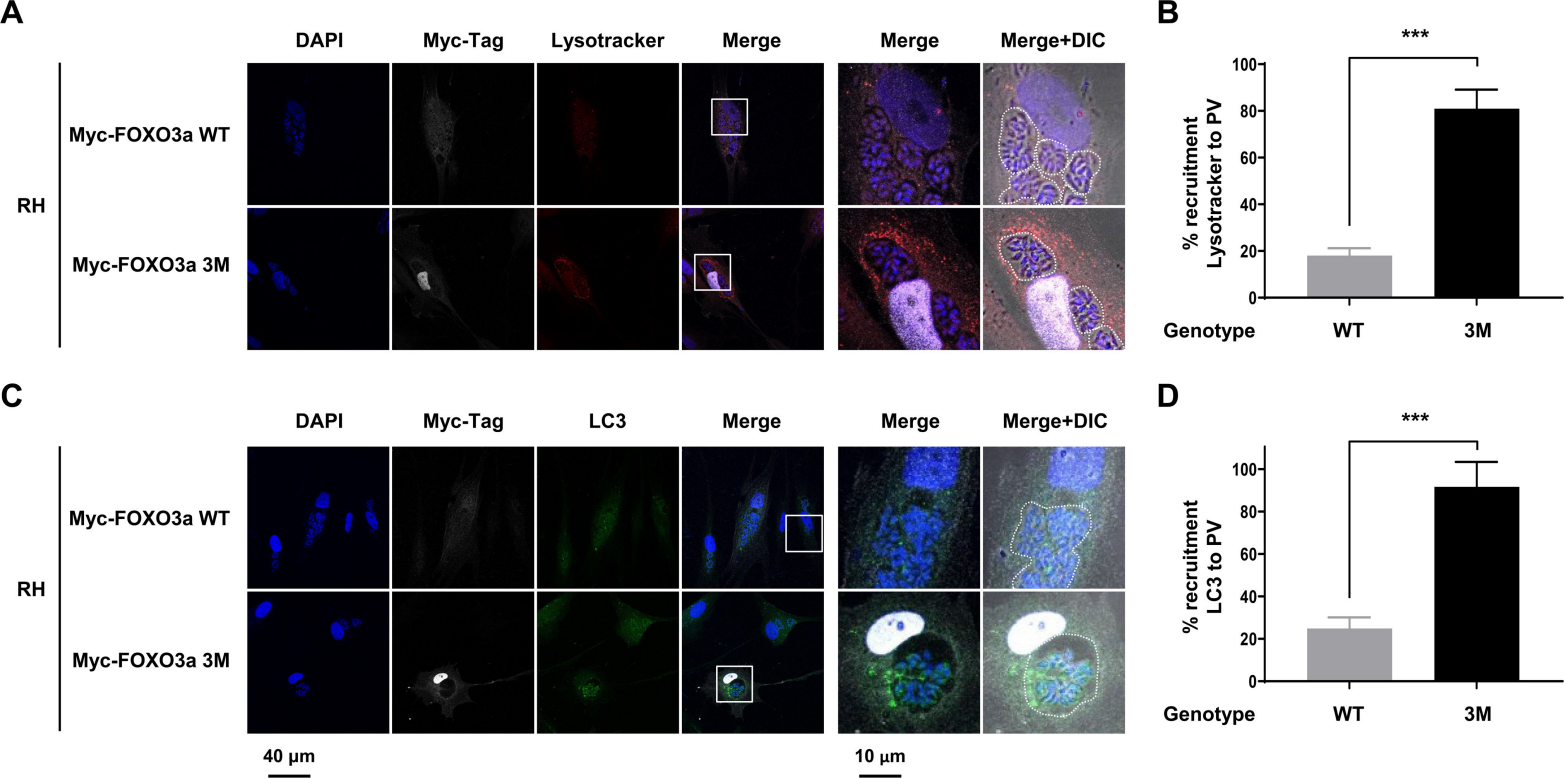
Recruitment of autophagy-related effectors to the parasitophorous vacuole is, in part, dependent on FOXO3a activity. (A, B) HFF were transduced to express either N-terminal Myc-tagged FOXO3a WT or TM forms. Cell cultures were inoculated with RH *T. gondii* parasites or left uninfected for 24 h. Cultures were deprived of FBS (i.e., starved) for the entire length of the experiment. Samples were processed for confocal immunofluorescence microscopy. Cells were stained with DAPI, for Myc-tagged FOXO3a, and (A, B) LysoTracker Red DND-99 (2 h prior to fixation) or (C, D) LC3. Original magnification (left panels) and four times-enlarged insets (right panels). Data are representative of two biological replicates. (A, C) PVs are outlined with dashed lines to indicate the presence of parasites within infected cells. Recruitment of (B) (Lysotracker-stained) and (D) LC3 to the PV was quantified. In Myc-tagged FOXO3a WT-expressing cells, 128 and 132 PVs were analyzed for lysosomal/autophagolysosomal structure and LC3 recruitment, respectively. In Myc-tagged FOXO3a TM-expressing cells, 99 and 118 PVs were analyzed for lysosomal/autophagolysosomal structure and LC3 recruitment, respectively. Data are presented as mean (SD) and are representative of three independent experiments (i.e., performed on different days). ***P < 0.0001.

Next, we compared the recruitment of acidic organelles and LC3 of the PV in HFF cells expressing either Myc-FOXO3a WT or Myc-FOXO3a TM variants. Myc-FOXO3a WT was readily exported from the nucleus upon *T. gondii* infection, while the recruitment of Lysotracker-positive structures and LC3 was minimal (Fig. 6A and C, top panels). These observations were in line with those made in WT HFF cultures (Fig. 5A and C, top panels). In stark contrast, mutation of the AKT-sensitive residues precluded nuclear export of the Myc-FOXO3a-TM form despite infection by *T. gondii* (Fig. 6A and C, bottom panels). Recruitment of autophagolysosomal structures and LC3 was markedly enhanced in infected cells expressing the TM form of FOXO3a (Fig. 6A and C, bottom panels, and Fig. 6B and D). These results were reminiscent of those obtained with the use of MK-2206 in WT HFF cells (Fig. 5A and C, bottom panels). Altogether, these data provide evidence that AKT-dependent nuclear export of FOXO3a is necessary to prevent the recruitment of autophagy-related effectors to the PV, a likely strategy by which *T. gondii* promotes its intracellular survival.

## DISCUSSION

Autophagy is a highly regulated catabolic process that targets cytosolic material for autophagolysosomal degradation (49). It has also evolved into an important host defense mechanism against viruses as well as intracellular bacteria and protozoan parasites such as *T. gondii* (9, 10). In this regard, it was previously described that CD40- and IFN-γ-mediated activation of autophagic response against *T. gondii* leads to parasite elimination (13, 14). However, *T. gondii* has developed several subversion strategies to counteract autophagic targeting (11, 15, 16, 48, 50, 51), including activation of host AKT signaling (15, 16). Despite this body of evidence, AKT downstream targets implicated in parasite-driven dysregulation of autophagy remained elusive. Herein, using cell imaging in combination with pharmacological and genetic approaches, we demonstrate that AKT-dependent curbing of the autophagic response by *T. gondii* depends, in part, on the inactivation of transcriptional programs controlled by FOXO3a.

Reduced nuclear FOXO3a levels were detected in murine macrophages infected with *T. gondii* (36). In line with these observations, we provide evidence that *T. gondii* actively forces FOXO3a out of the nucleus via AKT to hamper transcriptional programs involved in host defense responses in HFF. Similarly, the bacterium *C. rodentium* was shown to induce the translocation of nuclear FOXO3a into the cytosol of infected human HT-29 and mouse CMT-93 epithelial cell lines and colonic epithelium of infected mice (34). Even though no experimental evidence was provided on the alteration of transcriptional programs downstream of FOXO3a, the observed phenotype was associated with exacerbated pro-inflammatory cytokine production and disease pathogenesis during *C. rodentium* infection (34). We observed a dramatic increase in AKT-sensitive phosphorylation and nuclear exclusion of FOXO3a in *T. gondii*-infected HFF. In stark contrast, HCV was shown to induce FOXO3a phosphorylation at S574, a novel JNK-sensitive residue that promoted FOXO3a nuclear translocation in human hepatoma-derived HuH-7 cell line (52). These data hint at increased FOXO3a-mediated transcriptional activity; however, whether FOXO3a exerts a pro-viral or a host-protective role during HCV infection was not tested. These reports along with our data warrant further investigation on the biological consequences of pathogen-driven modulation of the phosphorylation and subcellular redistribution of FOXO3, two events that appear to be context-dependent.

FOXO3a subcellular localization and transcriptional activity do not seem to be modulated in response to other infectious agents such as *M. tuberculosis*, rhinovirus, and HIV; however, forward and reverse genetics approaches have provided evidence that FOXO3a-dependent transcriptional programs play a crucial role in the outcome of these infections (31 - 33). For instance, KD of FOXO3a led to non-protective responses in *M. tuberculosis*-infected macrophages, whereas overexpression of a constitutively active form of FOXO3a (i.e., FOXO3a TM) had the opposite effect (32). Likewise, conditional KO of FOXO3a in epithelial airway cells prevented type I and III IFN production and efficient antiviral immune responses in mice infected with rhinovirus (31). The same phenotype was reported in a stable FOXO3a KO human airway epithelial cell line (31). Interestingly, overexpression of FOXO1, another member of the FOXO family of transcription factors, restored the expression of Fas-associated factor 1, prevented IRF3 nuclear translocation, and abrogated interferon-stimulated gene expression in human epithelial cells infected with *T. gondii* (53), suggesting a role for FOXO1 in the regulation of IFN-mediated responses during toxoplasmosis. In addition, AKT-sensitive phosphorylation at T24 and PI3K-dependent nuclear exclusion of FOXO1 were recently reported in *T. gondii*-infected macrophages (36). However, it remains to be established whether *T. gondii* alters FOXO1 transcriptional programs in the host cell. Future work is required to shed light on the regulation and the biological consequences of FOXO1 nuclear exclusion during *T. gondii* infection.

Our data indicate that AKT and AKT-sensitive phosphorylation of FOXO3a in *T. gondii*-infected HFF require intact PI3K activity. These observations are in line with previous reports showing that either chemical blockade or genetic ablation of PI3K, using LY294002 or p110α siRNA, respectively, prevents AKT phosphorylation by *T. gondii* in both hematopoietic and non-hematopoietic human cells [i.e., monocytic THP-1 cells, primary brain microvascular endothelial cells (HBMEC), and human retinal pigment epithelial cells ARPE-19] (16, 40, 41). Activation of AKT at early stages of infection in HBMEC was shown to be triggered via EGFR autophosphorylation by *T. gondii* microneme (MIC) proteins MIC3 and MIC6, both adhesins harboring EGF‐like domains (16). Conversely, we did not detect early activation of EGFR-AKT signaling and AKT-mediated phosphorylation of FOXO3a in *T. gondii*-infected HFF. Prolonged PKCα/PKCβ-Src-dependent phosphorylation of EGFR has been implicated in sustained activation of AKT in *T. gondii*-infected human retinal pigment epithelial cells ARPE-19 (15). In stark contrast, HFF treatment with PKC and EGFR inhibitors (AG1418 and Gö6976, respectively) did not prevent prolonged phosphorylation of AKT and FOXO3a upon *T. gondii* infection. These reports, along with our data, suggest that *T. gondii* infection drives the activation of PI3K-AKT signaling through diverse mechanisms which appear to be, in part, time- and host cell type-dependent. Comparative *in cellulo* analyses and *in vivo* studies will shed light on the molecular underpinnings of the PI3K-AKT-FOXO3a axis during *T. gondii* infection in different cell types and tissues.

Pharmacological approaches employed in the present study also revealed that infection-induced phosphorylation of AKT and FOXO3a in HFF do not require mTOR activity. These data are reminiscent of those obtained in LPS-stimulated macrophages showing that, unlike PI3K, mTOR is dispensable for AKT-dependent phosphorylation of FOXO3a (17). Furthermore, AKT activation appeared to require infection by live parasites in HFF since treatment with STAg or HK parasites failed to trigger AKT-sensitive phosphorylation of FOXO3a. This latter observation does not exclude the possibility that secreted virulence factors or other soluble factors are linked to AKT and FOXO3a phosphorylation but rather that certain events are required for these factors to mediate their effects within the host cell (e.g., formation and presence of the PV membrane and specific route of entry of these molecules). Interestingly, both type I and II *T. gondii* strains tested (i.e., RH and ME49, respectively) were able to modulate FOXO3a phosphorylation and nuclear translocation in an AKT-dependent fashion, suggesting that this is a core process favoring parasite persistence that does not depend on strain-specific virulence factors. Taken together, our results indicate that phosphorylation and subsequent inactivation of FOXO3a by *T. gondii* require live infection and occur in a PI3K-AKT-dependent fashion independently of EGFR, PKCα, and mTOR activity in HFF. Further investigation will enable the identification of potential host and/or parasite factors involved in sustained AKT activation and subsequent nuclear exclusion of FOXO3a.

**Fig 7:**
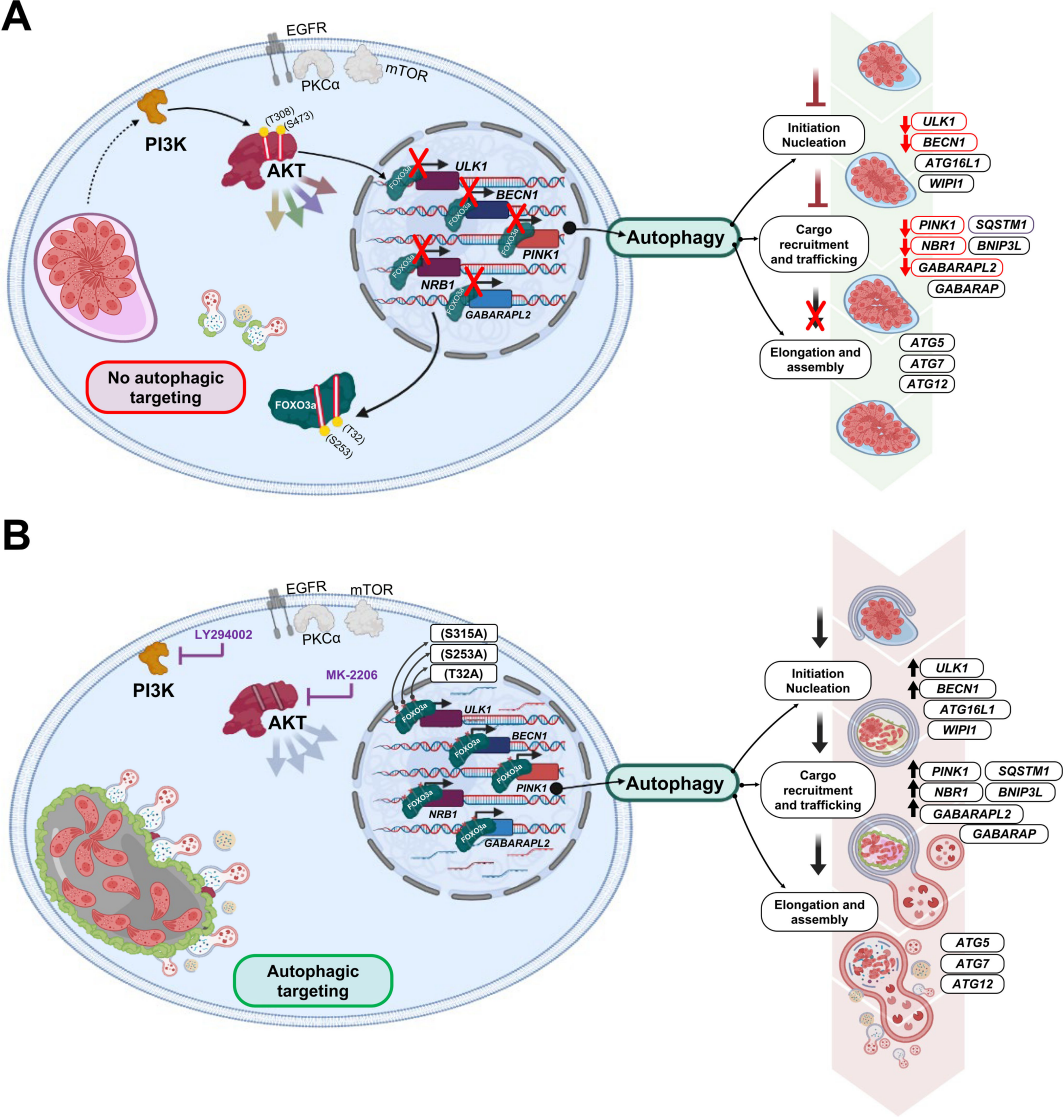
*T. gondii* represses FOXO3a-driven transcriptional programs to hamper autophagic targeting of the PV (Proposed Model). (A) Upon establishment and replication within the PV, *T. gondii* tachyzoites (shown in pink forming a rosette) activate the host cell PI3K-AKT signaling pathway independently of EGFR, PKCα, and mTOR. Phosphorylation of AKT (S473 and T308) leads to its activation and in turn to the phosphorylation of FOXO3a at AKT-sensitive residues (S253 and T32). Phosphorylation of FOXO3a at these residues leads to its nuclear exclusion and inactivation. As such, transcription of a subset of FOXO3a-dependent autophagy-related genes (i.e., *ULK1*, *BECN1*, *NBR1*, *PINK1*, and *GABARAPL2*) is downregulated (as indicated by the red “X” and the downward red arrows). Proteins encoded by this subset of transcripts are reported to participate in distinct steps of the autophagic response (right panel). Consequently, autophagic targeting of the PV is prevented, favoring parasite survival and replication. (B) Pharmacological inhibition of the PI3K-AKT pathway (i.e., treatment with LY294092 or MK-2206) precludes AKT-dependent phosphorylation and nuclear export of FOXO3a, thus promoting the transcription of autophagy-related genes (as indicated by upward black arrows) despite infection by *T. gondii*. Exogenous expression of an AKT-resistant form of FOXO3a harboring phosphosite mutations (S253A, T32A, and S315A) phenocopies chemical activation of FOXO3-driven autophagic targeting of *T. gondii*.

Our results are in agreement with two independent studies showing that inhibition of AKT signaling dramatically reduces *T. gondii* replication but does not hinder infection rates (16, 54), hinting at a crucial role for AKT activity to evade host cell defense mechanisms triggered after parasite internalization. Accordingly, intact PI3K-AKT signaling was identified as an essential mechanism utilized by *T. gondii* to hamper oxidative stress responses (54) and autophagy-mediated parasite clearance (16). Confirming and extending the latter report, we provide evidence that FOXO3a represents a downstream effector of AKT targeted by *T. gondii* to prevent accumulation of the autophagy protein LC3 and recruitment of acidic organelles around the PV. This is achieved, in part, through transcriptional repression of a subset of autophagy-related genes previously identified as *bona fide* FOXO3a targets using a combination of chromatin immunoprecipitation-Seq and RNA-Seq analyses (18). Parasite-directed silencing of FOXO3a-regulated autophagy transcripts identified in our screening may impede several steps of the host autophagic response, including initiation and nucleation and cargo recruitment (i.e., *BECN1* and *ULK1*) and trafficking (e.g., *NBR1* and *GABARAPL2*) (Fig. 7) (18, 30). In line with this notion, KD of *BECN1* in *T. gondii*-infected cells was shown to abrogate CD40-induced autophagic targeting of the PV (13) and prevent parasite killing upon chemical blockade of AKT signaling (16). Similarly, KD of *ULK1* restored parasite replication despite pharmacological downregulation of sustained AKT phosphorylation during *T. gondii* infection using an EGFR tyrosine kinase inhibitor (15). Further supporting our hypothesis that downregulation of FOXO3a transcriptional programs contributes to the multifaceted strategy utilized by *T. gondii* to stave off autophagic targeting, recruitment of LC3 and acidic organelle structures to the PV was markedly increased in cells expressing a mutated AKT-resistant form of FOXO3a. These observations are consistent with previous studies showing that overexpression of FOXO3a TM increases the number of LC3 puncta and promotes autophagic activity in microglia and HEK293T cells (17, 19). It is noteworthy that *T. gondii* manipulates autophagy through the positive and negative regulation of the autophagy effectors ATG5, ATG12, and ATG7 to hinder cargo-recruitment and elongation steps around the PV, thereby promoting parasite replication (50). Importantly, our data indicate that not all *bona fide* autophagy-related FOXO3a-regulated genes are modulated following *T. gondii* infection, while other genes, such as *SQSTM1*, seemed to be AKT-dependent but FOXO3a-independent. Therefore, *T. gondii*-driven transcriptional reprogramming of host autophagy genes cannot be solely attributed to FOXO3a dysregulation, and further investigation is required to identify additional players.

In sum, we report a novel mechanism employed by *T*. gondii to inhibit autophagic targeting through repression of FOXO3a transcriptional activity (Fig. 7). In recent years, promoting host cell autophagy has become an increasingly attractive therapeutic strategy against *T. gondii* (11). Interestingly, in addition to autophagy, other cellular processes that are transcriptionally regulated by FOXO3a (e.g., apoptosis, cell cycle, and oxidative stress) (22, 55) are also targeted by *T. gondii* (56). Moreover, FOXO3a has emerged as promising druggable target for various pathological conditions (e.g., cancer, diabetes, cardiovascular disease, and chronic neurological diseases) (22). Hence, it is tempting to speculate that restoring transcriptional programs regulated by FOXO3a, including but not limited to autophagy-related genes, could represent a new therapeutic approach to treat toxoplasmosis. Further characterization of altered transcriptional networks under the control of FOXO3a, and potentially other FOXO family members, during *T. gondii* infection will yield invaluable health-related knowledge to develop effective and safe host-directed strategies for better treatment or prevention of toxoplasmosis and potentially other infectious diseases.

## MATERIALS AND METHODS

### Reagents

Culture media and supplements were purchased from Wisent (St-Jean-Baptiste, Quebec, Canada) and Gibco (Grand Island, NY, USA); AG1478, Gö6976, and MK-2206 were obtained from Cayman Chemical (Ann Arbor, MI, USA); LY294002 was purchased from Chemdea (Ridgewood, NJ, USA); rapamycin was obtained from LC Laboratories (Woburn, MA, USA); and rhEGF was a gift from Dr. Stéphane Lefrançois (INRS—Centre AFSB, Laval, QC, Canada).

### Parasite maintenance and harvest

*T. gondii* tachyzoite cultures (RH and ME49 strains) were maintained by serial passages in Vero cells grown in DMEM culture medium supplemented with 5% heat-inactivated FBS, 2 mM l-glutamate, 1 mM sodium pyruvate, 100 U/mL penicillin, and 100 µg/mL streptomycin and incubated at 37°C, 5% CO_2_, as previously described (46). For experimental infections, freshly egressed tachyzoites were harvested from Vero cultures, pelleted by centrifugation (1,300 × *g*, 7 min, 4°C), resuspended in ice-cold PBS (pH 7.2–7.4), and passed through a syringe fitted with a 27 G needle. Large cellular debris and intact host cells were pelleted by low-speed centrifugation (200 × *g*, 3 min, 4°C), and the supernatant containing parasites was filtered with a 3-µm polycarbonate filter (Millipore, Burlington, MA, USA). Tachyzoites were then washed twice in PBS and finally resuspended in the appropriate culture medium according to the experiment.

### Soluble *T. gondii* antigens (STAg) and heat-killed (HK) parasites

STAg were prepared from freshly egressed tachyzoites, as previously described (57). Briefly, parasites were resuspended in ice-cold PBS, subjected to three 5-min cycles of freezing/thawing using liquid nitrogen and a 37°C water bath, and then sonicated on ice for 5 min (1 s on/off pulses, 30% duty cycle) using a Sonic Dismembrator FB505 (ThermoFisher, Waltham, MA, USA). Lysates were cleared by centrifugation (21,000 × *g*, 15 min, 4°C), and soluble material containing STAg was used for downstream experiments. HK parasites were prepared by incubating freshly egressed tachyzoites at 56°C for 10 min. After incubation, parasites were pelleted by centrifugation (1,300 × *g*, 7 min, RT) and resuspended in the appropriate culture medium according to the experiment.

### Infection of HFF and 3T3 fibroblasts

HFF and 3T3 cultures were plated one day before infection in DMEM culture medium supplemented with 10% heat-inactivated FBS, 2 mM l-glutamate, 1 mM sodium pyruvate, 100 U/mL penicillin, and 100 µg/mL streptomycin at 37°C, 5% CO_2_. Cultures were serum-starved for 1 h and treated with inhibitors when applicable. HFF cultures were then inoculated with live or HK parasites, treated with STAg, or left uninfected in fresh medium with 1% FBS unless otherwise indicated. Any remaining extracellular parasites were rinsed away with warm PBS (pH 7.2–7.4) 1 h following inoculation, fresh medium was added with inhibitors when applicable, and cells were incubated until the end of the experiment. When needed, cultures were deprived of FBS (i.e., serum-starved) for the entire length of the experiment to induce autophagy.

### Measurement of infection rates by flow cytometry

Parasite infection rates were determined by flow cytometry, as described (58). In brief, HFF cultures were pre-treated with MK-2206 or an equivalent volume of DMSO, then inoculated with *T. gondii* tachyzoites previously stained with 20 µM CellTracker Red (CMPTX) (Invitrogen, Waltham, MA, USA). Cultures were harvested at the indicated times by trypsinization, stained for 30 min at RT with the viability dye Live-or-Dye 750/777 (Biotium, Fremont, CA, USA), washed twice with FACS Buffer (PBS pH 7.2–7.4, 0.1% BSA), and then fixed with 1% paraformaldehyde (PFA) in PBS (15 min, on ice). Samples were analyzed by flow cytometry using a BD Fortessa (BD Biosciences, Franklin Lakes, NJ, USA), and downstream analyses were performed with FlowJo (BD Biosciences).

### Measurement of parasite replication

Parasite replication was evaluated by epifluorescence microscopy. Briefly, infected HFF cultures were fixed at the indicated times with PBS with 3.7% PFA (15 min, RT). Cells were permeabilized with PBS with 0.2% Triton X-100 (5 min, RT), stained with DAPI (5 min, RT), and then mounted onto slides. The number of parasites in at least 50 vacuoles in different fields for each time point and treatment was counted by microscopy using a 40× oil-immersion objective.

### Viability assays

Viability of HFF cultures was determined by the resazurin assay as described (59). Briefly, cells were treated with increasing concentrations of AG1478 (0.0625–8 µM), LY294002 (1.25–160 µM), MK-2206 (0.125–16 µM), Gö6976 (0.0625–8 µM), rapamycin (25–160 nM), or an equivalent volume of DMSO (vehicle) for 24 h at 37°C, 5% CO_2_. The medium was removed and replaced with fresh culture medium supplemented with 0.025% resazurin. Cultures were incubated for 4 h in the presence of the inhibitors or DMSO at 37°C, 5% CO_2_. Optical density was measured using a Multiskan GO (ThermoFisher) at 600 and 570 nm. Absorbance at 600 nm was subtracted from readings at 570 nm. Experiments were performed in biological replicates (*n* = 2); each sample was analyzed in a technical triplicate, the average of which was plotted against increasing concentrations of the respective inhibitor.

### Western blot analysis

Following infection and other treatments, cultures were lysed directly with pre-warmed Laemmli loading buffer diluted in RIPA lysis buffer (25 mM Tris [pH 7.6], 150 mM NaCl, 1% Triton-X 100, 0.5% sodium deoxycholate, 0.1% SDS) supplemented with complete EDTA-free protease inhibitor and PhosSTOP phosphatase inhibitor tablets (Sigma-Aldrich, St. Louis, MO, USA). Lysates were immediately heated at 95°C for 5 min and then stored at −80°C or processed immediately for SDS-PAGE. Resolved proteins were transferred onto PVDF membranes. Membranes were blocked for 1 h at RT in TBS 0.1% Tween-20 (TBS-T), 5% skim milk and then probed with the following primary antibodies: anti-phospho-EGFR (Y1068) (clone D7A5, #3777), anti-EGFR (clone D38B1, #4267), anti-phospho-AKT (S473) (clone D9E, #4060), anti-phospho-AKT (T308) (clone C31E5E, #2965), anti-AKT (clone C67E7, #4691), anti-phospho-FOXO3a (S253) (clone D18H8, #13129), anti-phospho-FOXO3a (T32) (polyclonal, #9464), anti-FOXO3a (clone 75D8, #2497), and β-actin (clone 8H10D10, #3700) were obtained from Cell Signaling Technologies (Danvers, MA, USA); anti-*T*. *gondii* profilin-like protein (polyclonal, #AF3860) was purchased from R&D Systems. Membranes were then probed with goat anti-rabbit, goat anti-mouse (Sigma-Aldrich), or rabbit anti-goat (R&D Systems, Minneapolis, MN, USA) IgG horseradish peroxidase-linked antibodies. Subsequently, proteins were visualized using the Clarity ECL Western blotting substrate (Bio-Rad, Hercules, CA, USA) and exposing the membranes to autoradiography film.

### Immunofluorescence and confocal microscopy

HFF were seeded onto glass coverslips in 24-well plates overnight. When applicable, cells were preloaded with the lysomotropic agent LysoTracker Red DND-99 (Invitrogen) diluted in DMEM (0.5 µM, final concentration) for 2 h at 37°C. At each time point, cells were rinsed with PBS three times and then fixed with 3.7% PFA in PBS for 15 min at RT. Cells were permeabilized with 0.2% Triton X-100 (in PBS) for 5 min at RT. Samples were kept in blocking solution (5% skim milk, 1% BSA, 5% normal goat serum in PBS) for 30 min at RT and incubated for 2 h at RT with the following primary antibodies diluted in PBS with 1% BSA: anti-FOXO3a (clone 75D8, #2497) and anti-Myc-Tag (clone 71D10, #2278) were obtained from Cell Signaling Technologies; anti-LC3 (clone 4E12, #M152-3) was purchased from MBL International (Woburn, MA, USA). Samples were then incubated with the following fluorochrome-conjugated secondary antibodies for 1 h at RT: goat anti-rabbit IgG (H + L) Alexa Fluor 647 (#A32733), donkey anti-rabbit IgG (H + L) Alexa Fluor 647 (#A-31573), and donkey anti-mouse IgG (H + L) Alexa Fluor 488 (#A-21202) were purchased from Invitrogen. Nuclei were stained with 300 nM 4',6-diamidino-2-phenylindole dilactate (DAPI) (Invitrogen) for 5 min at RT. Coverslips were mounted onto slides with Fluoromount G (Southern Biotech, Birmingham, AL, USA). Samples were visualized with the 40× objective of an LSM780 Zeiss confocal microscope (Oberkochen, BW, Germany), image acquisition was carried out using ZEN software, and image processing was performed with Icy Software from the Institut Pasteur (60).

### Quantitative RT-PCR

RNA was extracted with Qiazol (Qiagen, Hilden, NRW, Germany) according to the manufacturer’s specifications. Purified RNA (500 ng) was reverse transcribed using LunaScript RT SuperMix Kit (New England Biolabs). Quantitative PCR was performed with Luna qPCR Master Mix (New England Biolabs). Relative quantification was calculated using the comparative Ct method (ΔΔCt) (61), and relative expression was normalized to human *ACTB*. Experiments were performed in independent biological replicates (*n* = 3); each sample was analyzed in a technical triplicate, the average of which was plotted against the respective conditions used. Primers were designed using NCBI Primer-BLAST (http://www.ncbi.nlm.nih.gov/tools/primer-blast/) (Table S1).

### Lentivirus production and HFF transduction

Lentiviruses were produced in HEK293T cells using Lenti-Pac HIV Expression Packaging Kit, as per manufacturer’s guidelines (GeneCopoeia, Rockville, MD, USA; #LT001). Lentivirus titers were measured using Lenti-Pac HIV qRT-PCR Titration Kit (GeneCopoeia, #LT001) according to the manufacturer’s protocol. HFF cells were transduced with the different lentivirus preparations for 3 days in the culture medium supplemented with 5 µg/mL Polybrene (hexadimethrine bromide). Transduced cultures were either used immediately for downstream experiments or selected for 6 days using 2 µg/mL puromycin to generate stable cell lines. Plasmids, shRNA clones, and open reading frame expression clones were purchased from GeneCopoeia (Table S2).

### Statistical analysis

Where applicable, data are presented as the mean (standard deviation) (SD) of the mean. Statistical significance was determined by using one-way ANOVA followed by a Tukey *post hoc* test or a two-tailed independent Student’s *t*-test followed by a Bonferroni *post hoc* test; calculations were performed by using Prism 7 software package (GraphPad). Differences were considered significant when *P < 0.05, **P < 0.01, ***P < 0.001, and ****P < 0.0001.

## Data Availability

All relevant data are within the manuscript and its supplemental material.

## SUPPLEMENTAL MATERIAL

Supplemental Figures and Tables mbio.00795-23-s0001.pdf Figures S1-S9. Tables S1-S2.

ASM does not own the copyrights to Supplemental Material that may be linked to, or accessed through, an article. The authors have granted ASM a non-exclusive, world-wide license to publish the Supplemental Material files. Please contact the corresponding author directly for reuse.
