# Supplementary material for: Toxoplasma gondii inhibits the expression of autophagy-related genes through AKT-dependent inactivation of the transcription factor FOXO3a
Source: mBio. 2023 Jun 30;14(4):e00795-23. doi: 10.1128/mbio.00795-23 (PMC10470550; doi:10.1128/mbio.00795-23)
Supplement: Supplemental Figures and Tables — Figures S1-S9. Tables S1-S2. [file mbio.00795-23-s0001.pdf]

Supporting information figures, tables, and captions

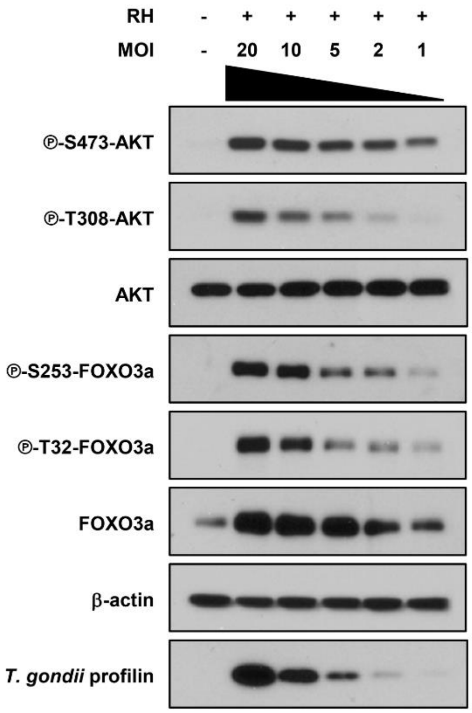

Figure S1. The multiplicity of infection (MOI) influences phosphorylation levels of AKT and FOXO3a in *T. gondii*-infected cells. HFF cultures were inoculated with RH *T. gondii* tachyzoites at the indicated MOI or left uninfected. Samples were collected 24 h after infection. Phosphorylation and expression levels of indicated proteins were monitored by western blotting. Total amounts of β-actin were used as a loading control, and an antibody raised against *T. gondii* profilin-like protein was used to assess infection of HFF cultures.

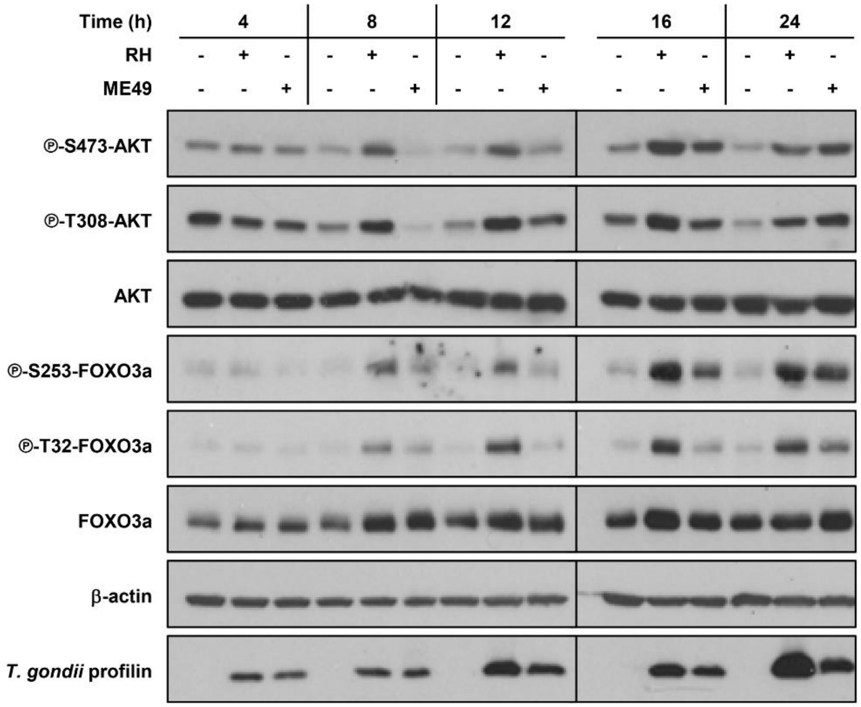

Figure S2. Infection of 3T3 by *T. gondii* induces phosphorylation of host AKT and FOXO3a

3T3 cultures were inoculated with either RH or ME49 *T. gondii* tachyzoites or left uninfected for the indicated times. Phosphorylation and expression levels of indicated proteins were monitored by western blotting.

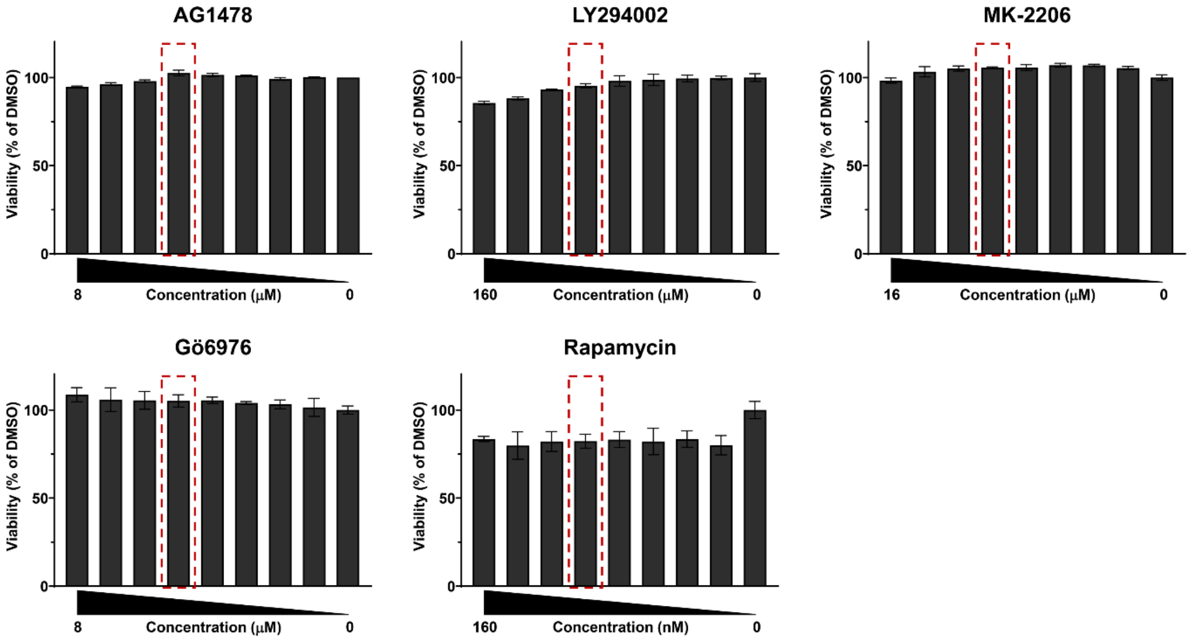

Figure S3. Selected inhibitors do not display acute toxicity in HFF cells. HFF cultures were treated with increasing (two-fold) concentrations of different inhibitors (AG1478, LY294002, MK-2206, Gö6976, and rapamycin) or an equivalent volume of vehicle (i.e., DMSO), as indicated, for 32 h. Cell viability was measured by colorimetric-based resazurin assays. The concentration used in subsequent experiments is identified by a red dashed box for each inhibitor. OD values were normalized to DMSO-treated samples. Results are presented as mean (SD); all samples were performed in technical triplicates. No statistically significant differences in cell viability were observed between the various inhibitors and their respective controls.

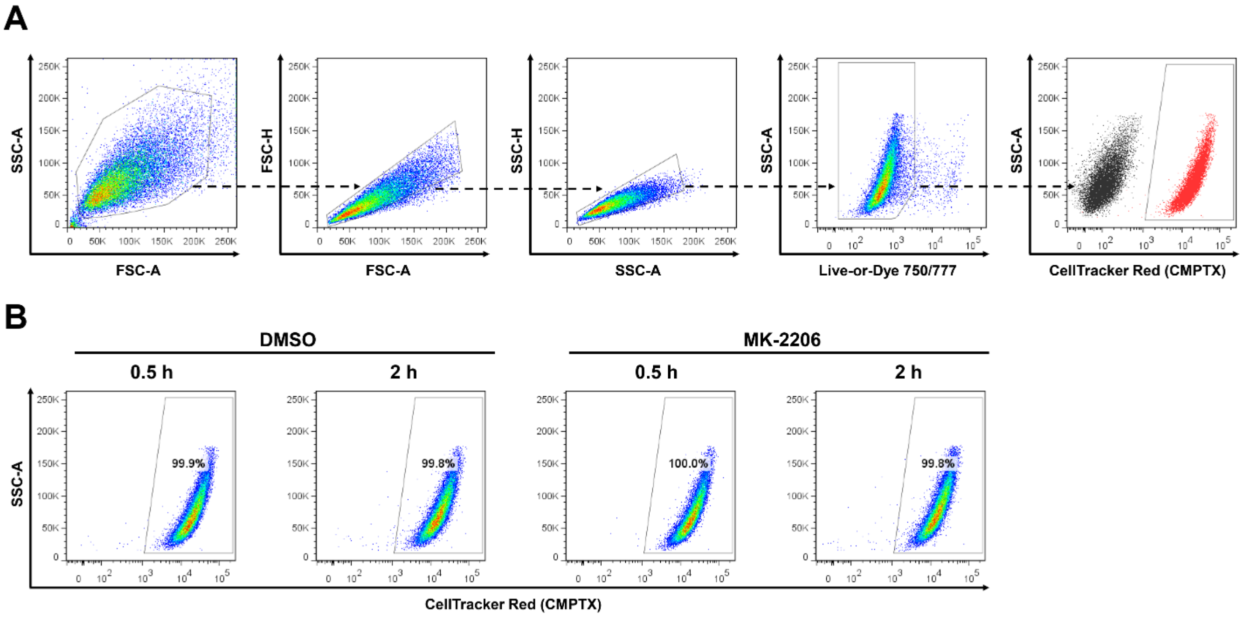

Figure S4. MK-2206 treatment of HFF does not affect infection rates by *T. gondii*.

(A, B) HFF cultures were pre-treated with 2 μM MK-2206 or an equivalent volume of vehicle (i.e., DMSO) for 1 h then inoculated with CellTracker Red (CMPTX)-stained RH *T.* gondii parasites. Cells were collected by trypsinization 0.5 and 2 h post-infection, then processed for flow cytometry analyses to monitor infection rates. **(A)** Shown here, gating strategy utilized to measure infection rates. First, cells were identified according to FSC-A and SSC-A scatter profiles. Then, singlets were gated based on FSC-A vs. FSC-H and SSC-A vs. SSC-H. Dead cells were gated out, and only live cells were considered according to low staining with Live-or-Dye 750/777. Infected cells were identified based on positive signal for CellTracker Red. (**B**) Infection rates for cultures reported for DMSO- (left panels) and MK-2206-treated (right panels) cells at the indicated timepoints. Data and data analyses are representative of two independent experiments (i.e., performed on different days).

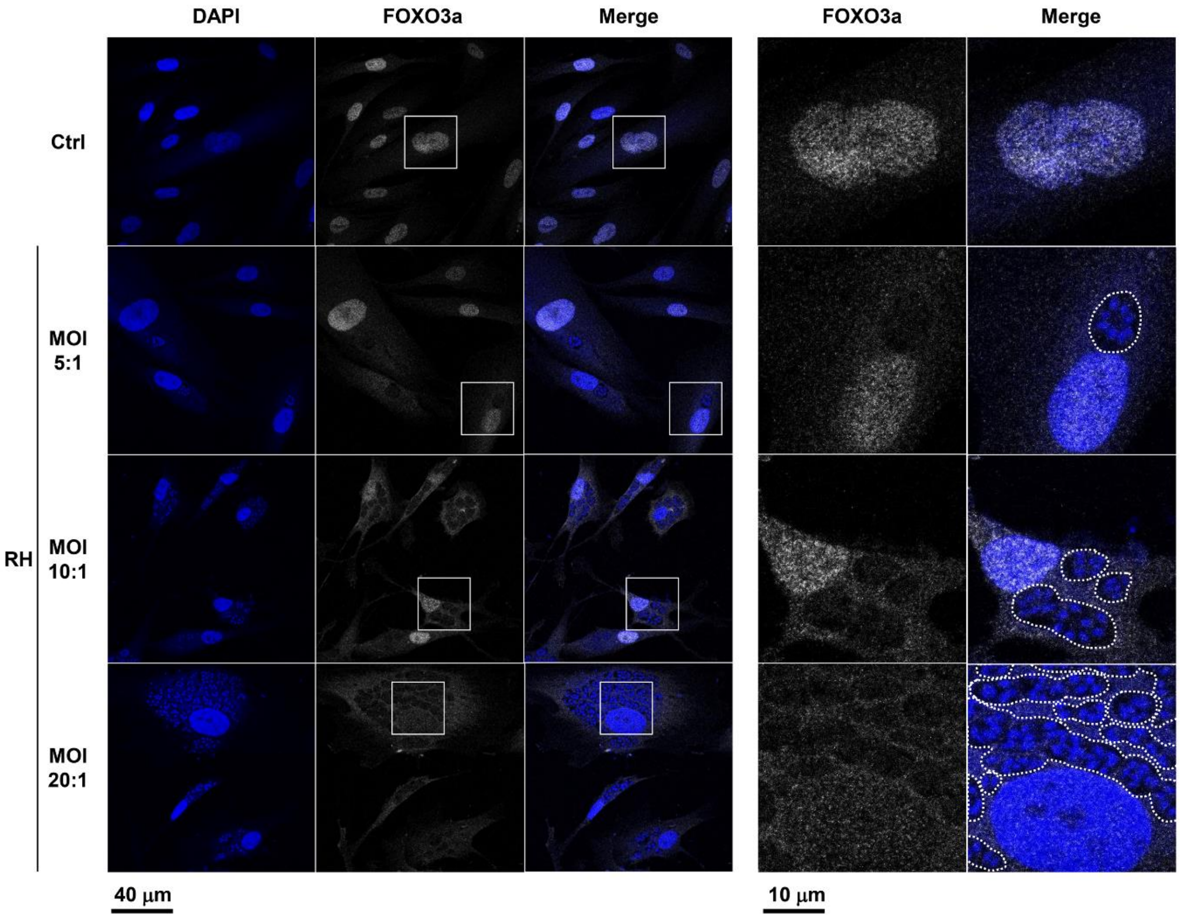

Figure S5. The multiplicity of infection (MOI) influences nuclear export of FOXO3a in *T. gondii*-infected cells. HFF cultures were inoculated with RH *T. gondii* tachyzoites at the indicated MOI or left uninfected. Cultures were fixed 32 h after infection and processed for confocal immunofluorescence microscopy. PVs are outlined with dashed lines to indicate the presence of parasites within infected cells. Data are representative of two independent experiments (i.e., performed on different days).

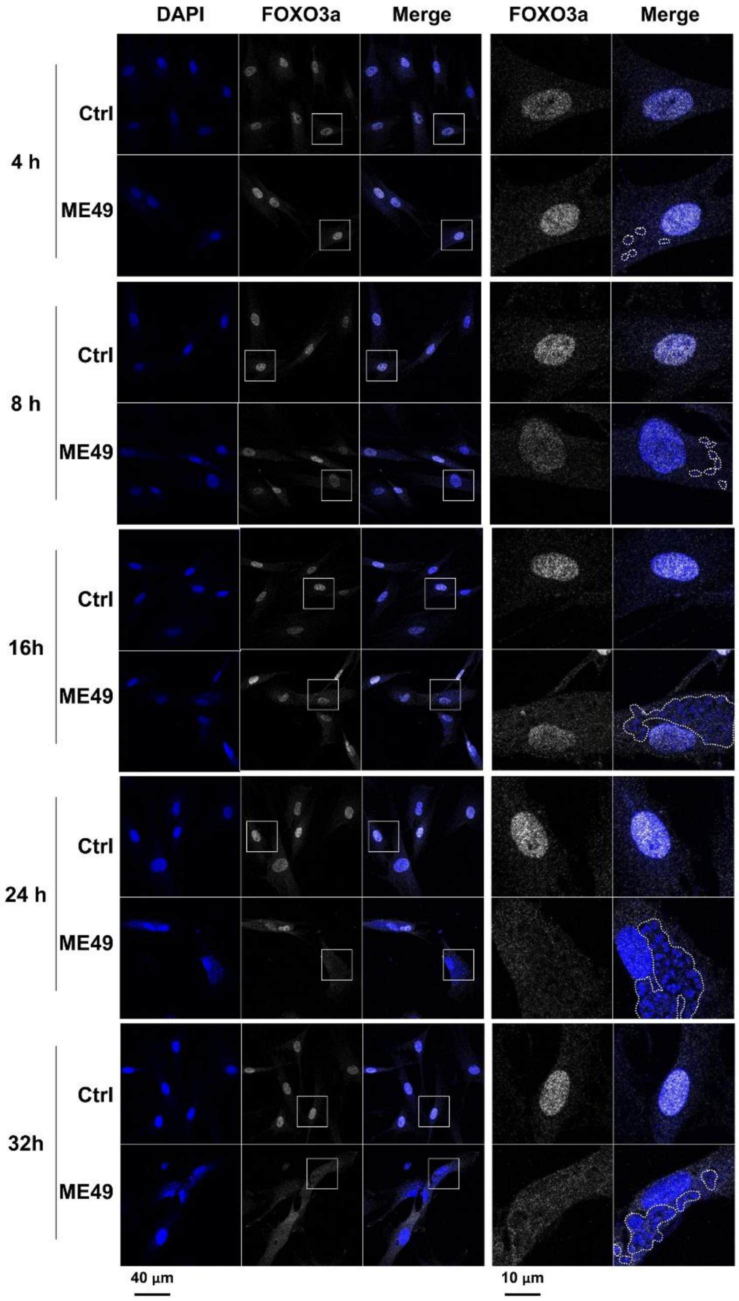

Figure S6. Infection with type II *T. gondii* strain ME49 leads to FOXO3a nuclear export from the host nucleus. HFF cultures were inoculated with ME49 *T. gondii* tachyzoites or left uninfected and fixed at the indicated times and processed for confocal immunofluorescence microscopy. Samples were stained with DAPI (shown in blue) and for total FOXO3a (shown in white). Original

magnification (left panels), 4 times-enlarged insets (right panels). PVs are outlined with dashed lines to indicate the presence of parasites within infected cells. Images are representative of two independent experiments (i.e., performed on different days).

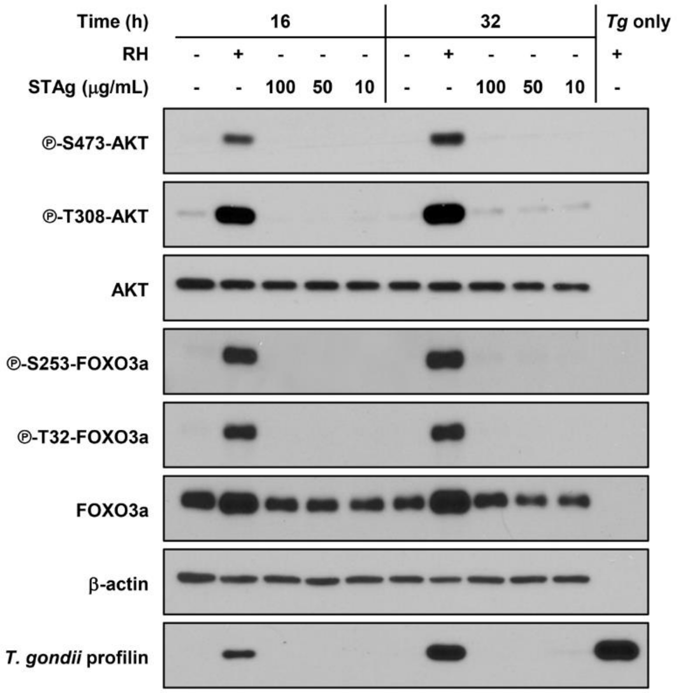

Figure S7. Soluble *T. gondii* antigens (STAg) fail to induce FOXO3a phosphorylation and phenocopy infection with live parasites. HFF cultures were inoculated with RH *T. gondii* tachyzoites, treated at the indicated STAg concentration, or left uninfected and untreated for the indicated times. Phosphorylation and expression levels of indicated proteins were monitored by western blotting.

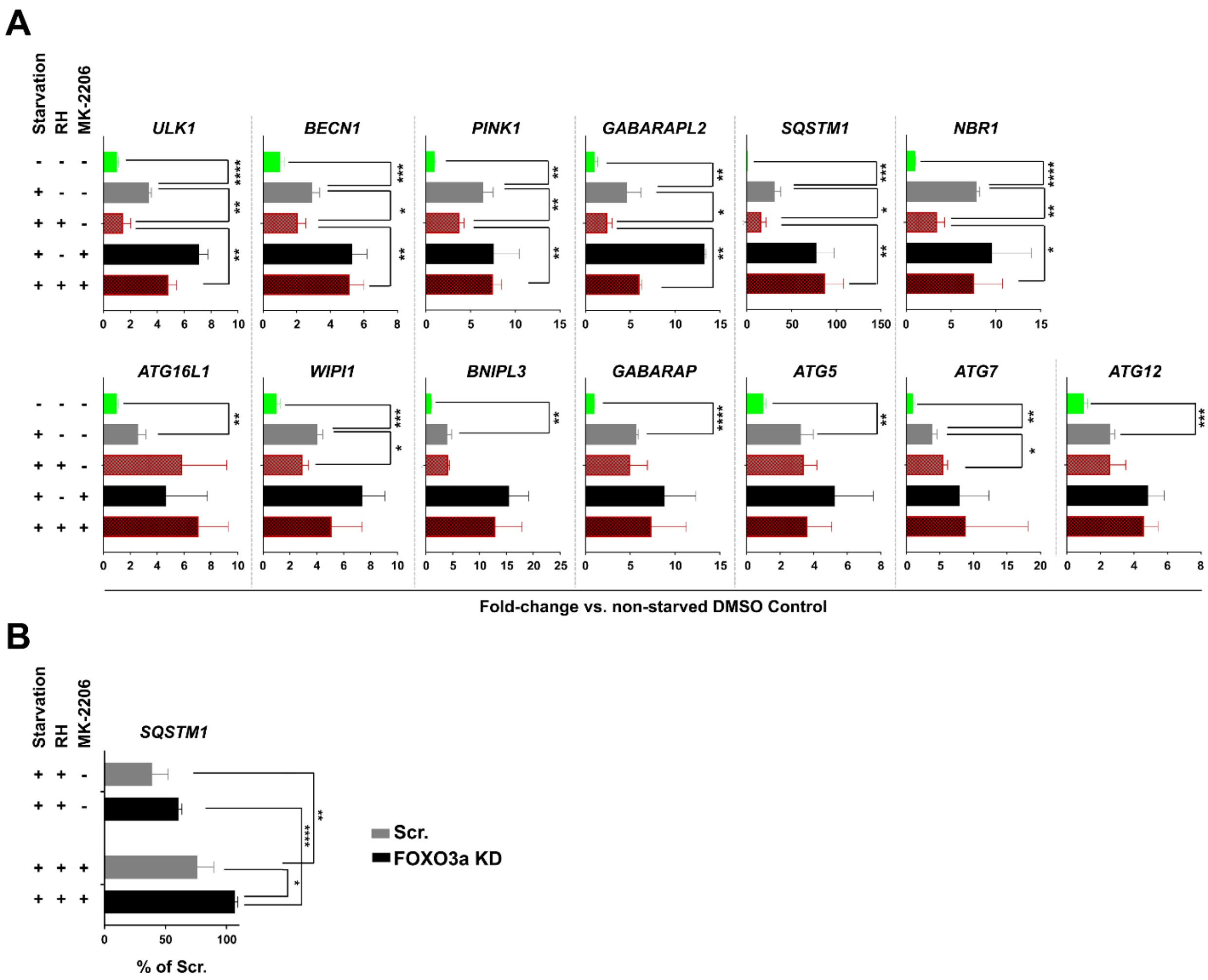

Figure S8. Infection by *T. gondii* inhibits serum starvation-induced expression of a subset of autophagy-related genes through AKT-FOXO3a-dependent mechanisms. (**A**) HFF cultures were pre-treated with 2 μM MK-2206 or an equivalent volume of vehicle (i.e., DMSO). Then, cells were inoculated with RH *T. gondii* tachyzoites or left uninfected for 24 h. Cultures were deprived of FBS (i.e., serum-starved) for the entire length of the experiment to induce autophagy or not, as indicated. **(A, B)** Samples were processed for qPCR analyses. Relative mRNA amounts of a subset of autophagy-related transcripts (normalized to *ACTB*) were compared to uninfected control cultures. Relative expression fold-change of the indicated genes was calculated against **(A)** non-starved DMSO-treated uninfected control or **(B)** as a percentage of serum-starved DMSO-

treated uninfected Scr. HFF cultures. Each sample was analyzed in technical triplicates, the averages (SD) of which are plotted. Data are representative of at least two independent experiments (i.e., performed on different days).

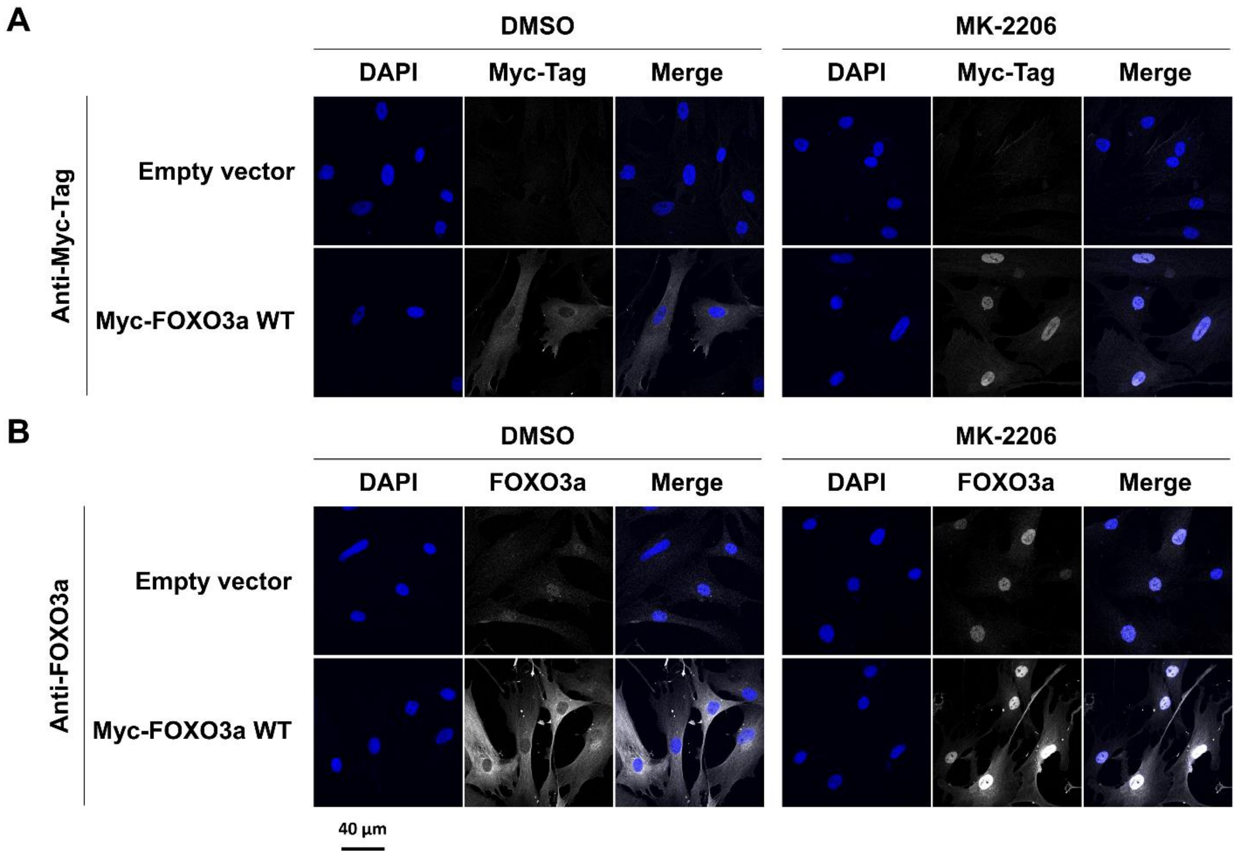

Figure S9. Exogenous Myc-FOXO3a WT protein behaves like the endogenous form and is sensitive to AKT activity. (A, B) HFF cultures were transduced to express an N-terminal Myc-tagged FOXO3a WT form or transduced with the empty vector. Cells were treated with 2 µM MK-2206 or an equivalent volume of vehicle (i.e., DMSO) for 4 h. Samples were processed for confocal immunofluorescence microscopy. Samples were stained with DAPI (shown in blue) and for Myc-tagged FOXO3a WT (shown in white) using anti-Myc-tag (**A**) or anti-FOXO3a (**B**) antibodies.

Table S1. List of primers used for RT-qPCR experiments

**Target**	**Sense**	**Sequence (5'→ 3')**
ACTB	Forward	TGACCCAGATCATGTTTGAGACC
	Reverse	AGGGATAGCACAGCCTGGAT
FOXO3	Forward	CGTGCCCTACTTCAAGGATAA
	Reverse	ATTCTGGACCCGCATGAATC
ATG5	Forward	CACAAGCAACTCTGGATGGGATTG
	Reverse	GCAGCCACAGGACGAAACAG
ATG7	Forward	TCGAAAGCCATGATGTCGTCTT
	Reverse	CCAAAGCAGCATTGATGACCA
GABARAP	Forward	CTCCCTTATTCAGGACCGGC
	Reverse	TGCCAACTCCACCATTAC
ATG12	Forward	TGCTAAAGGCTGTGGGAGAC
	Reverse	ACTGTTCTGAGGCCACAAGTT
ATG16L1	Forward	GCATGACGTACCAAACAGGC
	Reverse	ACTCCCCACGTTTCTTGTGT
BECN1	Forward	CCACAGAAAGTGCCAACAGC
	Reverse	GACGTTGAGCTGAGTGTCCA
BNIP3L	Forward	GGACTCGGCTTGTTGTGTTG
	Reverse	TCGACTAGGTGGGACGAC
GABARAPL2	Forward	AGTCCCACAGTCCAGCCTAA
	Reverse	CGCAAAAGTGTTCTCTCCGC
NBR1	Forward	ATTCACCCCACAGGGATAGC
	Reverse	AACCTGTGGTTCCATGCTGT
SQSTM1	Forward	TGTGTAGCGTCTGCGAGGGAAA
	Reverse	AGTGTCCGTGGTTCACCTTCCCG
PINK1	Forward	CCTGGAGTGTGAAACGCTCT
	Reverse	CTCCCACCCTCACCATTCAC
ULK1	Forward	GGACACCATCAGGCTCTTCC
	Reverse	GAAGCCGAAGTCAGCGATCT
WIPI1	Forward	TGCACATCCCTAGCAACTGG
	Reverse	CTCCACGATGTAGACGTCGC

Table S2. List of shRNA and ORF clones used for reverse- and forward-genetics experiments, respectively.

**shRNA clones**		
**Target**	**Catalog # Genecopoeia**	**Description**
Scramble (Scr.)	CSHCTR001-LVRU6MP	shRNA scrambled control clone for psi-LVRU6MP
FOXO3	CS-HSH005759-LVRU6MP-02	shRNA clone set of 3 constructs against 3 variants for human *FOXO3* (ENST00000540898.1, NM_001455.4, and NM_201559.3) in lentiviral psi-LVRU6MP vector with U6 promoter, mCherry, puromycin
**Vector and ORF clones**		
**Label**	**Catalog # Genecopoeia**	**Description**
Empty Vector	EX-NEG-Lv107	Empty control vector for pReceiver-Lv107 vector
Myc-FOXO3a WT	EX-Z1129-Lv107	ORF expression clone for human *FOXO3* (NM_201559.2) in lentiviral pReceiver-Lv107 vector with CMV promoter, N-Myc tag, puromycin
Myc-FOXO3a TM	CS-Z1129-Lv107-01	Custom ORF expression clone for human FOXO3 (NM_201559.2) with T32A, S253A & S315A mutations in lentiviral pReceiver-Lv107 vector with CMV promoter, N-Myc tag, puromycin
